# Trends in overweight prevalence among university freshmen and the impact of the COVID-19 pandemic: a repeated cross-sectional study

**DOI:** 10.3389/fpubh.2026.1856579

**Published:** 2026-07-01

**Authors:** Jiechen He, Xuan Yin, Jianguang Ji, Zhifeng Zhang

**Affiliations:** 1 Faculty of Health Sciences, University of Macau, Taipa, Macao SAR, China; 2 Public Physical Education Department, Hebei North University, Zhangjiakou, China

**Keywords:** body mass index, COVID-19 pandemic, interrupted time series analysis, overweight rate, university freshmen

## Abstract

**Background:**

Existing studies on body mass index (BMI) among university students have largely focused on short-term changes during the Coronavirus Disease 2019 (COVID-19) pandemic, with limited evidence on long-term trajectories across different pandemic phases. Therefore, this study aimed to evaluate trends in BMI and overweight prevalence among Chinese university freshmen from 2016 to 2024 using repeated cross-sectional data and to quantify both the immediate and sustained effects of COVID-19 policy transitions.

**Methods:**

Anthropometric data were collected from successive cohorts of freshmen from a Chinese university between 2016 and 2024. BMI was calculated and categorized into overweight and non-overweight groups. Long-term trends in BMI and overweight prevalence were assessed using linear and non-linear models. Interrupted time series (ITS) analysis was further applied to evaluate changes across the pre-, during-, and post-pandemic periods. Subgroup analyses were conducted by sex and academic discipline.

**Results:**

A total of 39,478 freshmen were included. Overall, a rising trend in BMI and overweight prevalence was observed among university students over the study period. Crude ITS analysis showed that BMI increased gradually before the pandemic (*β*_1_ = 0.096 kg/m^2^/year, *p* < 0.001), followed by a temporary slowdown during the pandemic period (*β*_4_ = −0.222 kg/m^2^/year, *p* < 0.001). In the post-pandemic period, BMI and overweight prevalence increased markedly, characterized by a significant upward slope change (*β*_5_ = 0.738 kg/m^2^/year, *p* = 0.001) reaching the highest levels in 2024. Males exhibited a higher prevalence of overweight and a steeper post-pandemic increase than females. Discipline-specific differences demonstrated that humanities, arts and social sciences (HASS) students had a more immediate change. In contrast, science, technology, engineering, and mathematics (STEM) students exhibited a more sustained increase over time.

**Conclusion:**

BMI trajectories were significantly altered by the pandemic, with a marked post-pandemic acceleration exceeding pre-existing trends. Future longitudinal studies are needed to clarify the behavioral mechanisms underlying these trends.

## Introduction

The global escalation of overweight represents one of the most formidable public health challenges of the 21st century. Recent data from the non-communicable disease (NCD) Risk Factor Collaboration (NCD-RisC) indicate that the number of people living with overweight worldwide has surpassed one billion, with the prevalence among adults having doubled since 1990 (1, 2). This pandemic of overweight carries profound clinical implications, as overweight is a major driver for a wide range of NCDs, including type 2 diabetes (3), cardiovascular diseases, and at least 13 types of cancer (4). Beyond morbidity, the mortality burden is substantial. High body mass index (BMI) contributes to millions of deaths globally each year and is associated with an increased risk of all-cause mortality as BMI rises into the obese range (5). In China, the burden of overweight and obesity has increased substantially over recent decades. Recent national estimates indicate that China has the largest population of overweight and obese individuals worldwide, with approximately 34.3% of adults classified as overweight, affecting more than 600 million people (6). In addition to its health consequences, excess body weight imposes a considerable economic burden. A nationally representative study estimated that overweight- and obesity-attributable healthcare expenditures reached approximately 24.35 billion RMB annually, accounting for 2.46% of total healthcare expenditure in China (7). More recent evidence further suggested that overweight and obesity accounted for approximately 3.9% of total healthcare costs nationwide (6). As these health and economic burdens continue to grow, understanding weight-related trends among young populations has become an important public health priority.

Of particular concern is the shift toward earlier onset of overweight, with increasing prevalence among adolescents and young adults leading to prolonged exposure to excess adiposity and earlier onset of chronic diseases (8, 9). The transition from high school to university represents a critical and vulnerable window for lifestyle restructuring. During this period, increased autonomy in dietary choices, reduced physical activity, and irregular sleep patterns may contribute to rapid weight gain, a phenomenon commonly referred to as the “Freshman 15” (10). Importantly, longitudinal evidence suggests that weight gain during the college years is rarely reversed, and it can predict overweight-related metabolic risks in later adulthood (10, 11). This trajectory was further complicated by the Coronavirus Disease 2019 (COVID-19) pandemic, which substantially disrupted campus life. Lockdown measures were associated with reduced physical activity, increased sedentary behavior, and heightened psychological stress among university students, all of which are well-established obesogenic factors (12). In China, these effects may have been particularly pronounced because of the implementation of the “Zero-COVID” strategy, which was among the most stringent and prolonged pandemic control policies worldwide (13, 14). Such measures may have further reinforced changes in students’ daily routines and health-related behaviors.

Importantly, behavioral patterns established during prolonged periods of restriction may persist beyond the relaxation of public health measures and gradually evolve into a “new normal” (15, 16). Consequently, health outcomes may not automatically return to pre-pandemic levels, highlighting the importance of examining post-pandemic trajectories. However, most studies rely on short-term or cross-sectional data and fail to capture long-term trajectories across the pre-, mid-, and post-pandemic periods. In addition, the post-pandemic phase, particularly potential rebound effects, remains underexplored, leaving uncertainty about whether student health has recovered or further deteriorated.

Additionally, academic discipline may reflect important differences in students’ academic demands, daily routines, and lifestyle behaviors (17). For example, students in science, technology, engineering, and mathematics (STEM)-related disciplines often experience intensive coursework, laboratory training, and prolonged screen time, whereas students in humanities and social sciences may have different study patterns and schedules. These discipline-specific characteristics may influence sedentary behavior, physical activity, sleep habits, dietary behaviors, and stress levels, all of which are associated with body weight and metabolic health. Therefore, examining BMI trajectories across academic disciplines may help identify whether the impact of the COVID-19 pandemic differed among student groups with distinct academic and lifestyle profiles.

Therefore, this study aims to evaluate temporal trends in BMI and overweight prevalence among university freshmen from 2016 to 2024, with a focus on changes across pre-, mid-, and post-pandemic periods. We further assess the immediate and sustained impacts of COVID-19 and explore heterogeneity by sex and academic discipline. We hypothesized that BMI and overweight prevalence would increase overtime among university students and the COVID-19 pandemic would disrupt these pre-existing trajectories. We further hypothesized that BMI and overweight prevalence would not fully return to pre-pandemic levels during the post-pandemic period, with potential variations across population subgroups.

## Methods

### Study design and data collection

This is a repeated cross-sectional study to analyze the temporal trends and impact of COVID-19 on BMI and overweight among university freshmen from 2016 to 2024. In China, universities implement a routine health examination system for newly admitted students. Each year, freshmen who are enrolled in universities undergo a physical examination in September, during which their height and weight are measured. These examinations are conducted by licensed medical institutions commissioned by universities, following standardized protocols. Anthropometric measurements are performed by trained healthcare professionals, and all measurements are recorded on site by examination nurses using standardized data collection forms, ensuring consistency and data quality across years (18).

Body height was measured using a standardized electronic height meter (measurement accuracy 0.1 cm). Body mass was measured using a calibrated digital scale (measurement accuracy 0.01 kg). Ethical approval for this study was provided by the Ethics Committee of Hebei North University (2026GT001). The requirement for informed consent was waived because the study used de-identified secondary data extracted from the university health examination database.

### Participants

**Figure 1:**
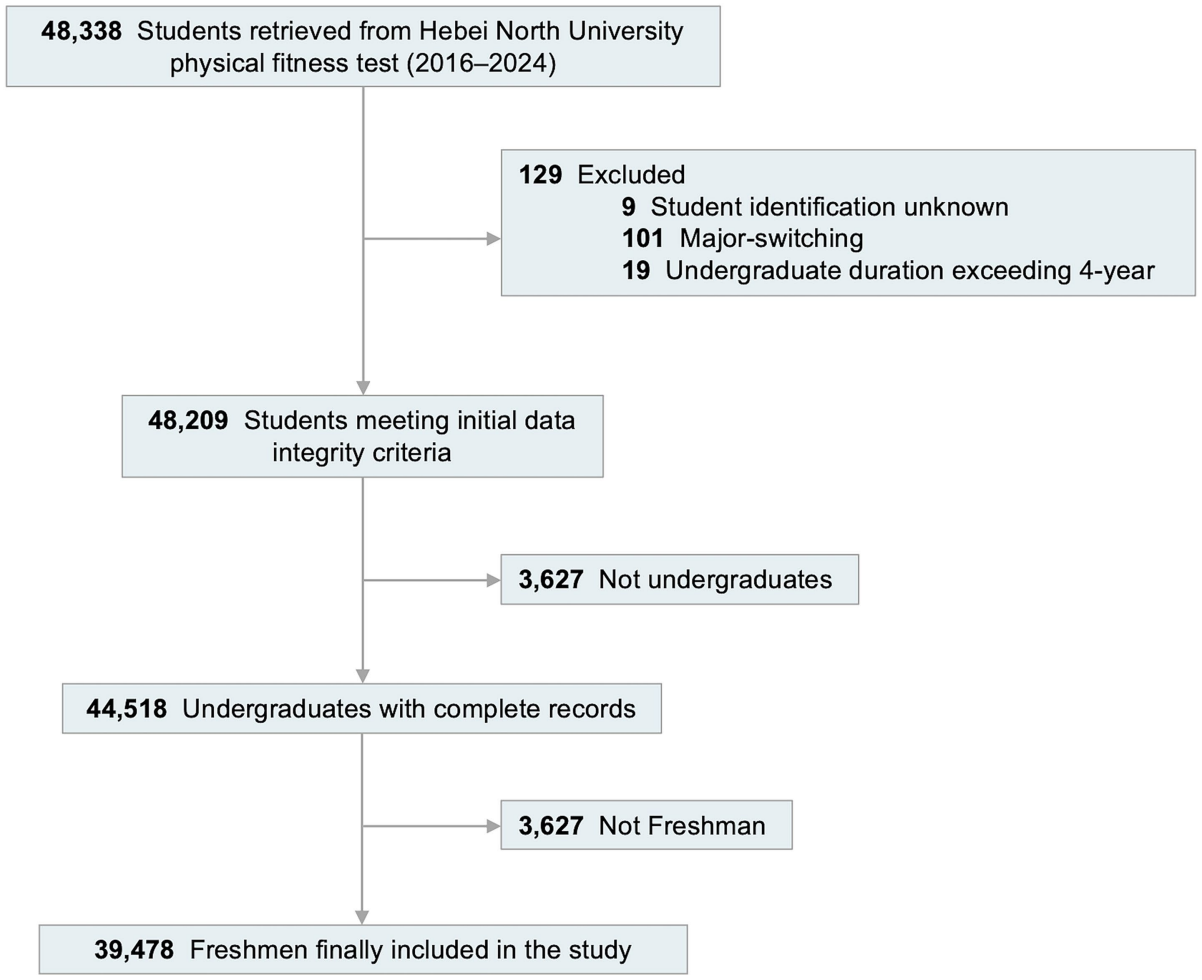
Flow chart of the study population.

Participants were initially retrieved from the physical fitness test database of Hebei North University between 2016 and 2024. Participants were included if they met the following criteria: (i) being enrolled as undergraduate freshmen between 2016 and 2024; (ii) possessing complete physical fitness test records conducted during the first semester of enrollment year. The physical fitness data for the 2021 freshmen, which could not be measured due to the COVID-19 pandemic, were treated as structurally missing (19). The exclusion criteria included: (i) students enrolled in non-baccalaureate programs (e.g., junior college programs that do not confer a formal degree); (ii) individuals with major-switching records, or an undergraduate duration exceeding 4 years; and (iii) participants who were not freshmen at the time of physical fitness test. A study flow chart is illustrated in Figure 1.

### Measurements of outcomes

Two outcomes were assessed in this study: BMI and overweight status. BMI was calculated as body weight in kilograms divided by the square of height in meters (kg/m^2^). Overweight status was defined as a binary outcome, with overweight classified as BMI ≥ 25 kg/m^2^, according to the World Health Organization (WHO) criteria to facilitate international comparisons (1). Considering the Chinese study population, we additionally repeated all analyses using the Working Group on Obesity in China (WGOC) criterion (BMI ≧ 24 kg/m^2^) as a sensitivity analysis to assess the robustness of the results (20).

### Classification of academic disciplines

Academic disciplines were classified according to the United Nations Educational, Scientific and Cultural Organization (UNESCO) International Standard Classification of Education Fields of Education and Training 2013 (ISCED-F 2013). Majors belonging to natural sciences, Information and Communication Technologies (ICT), engineering, agriculture/veterinary sciences, and health-related disciplines were categorized as STEM, whereas majors belonging to education, arts and humanities, social sciences, business and management, and service-related disciplines were categorized as humanities, arts and social sciences (HASS) (21). Detailed classifications are provided in Supplementary Table 1.

### Statistical analysis

Descriptive statistics were employed to describe participant characteristics. Continuous variables (e.g., BMI) were presented as mean and standard deviation (mean ± SD), while categorical variables (e.g., sex, major type, and overweight rate) were expressed as frequency and percentage (%).

Long-term trends in BMI and overweight rate were analyzed using both linear and non-linear modeling approaches. Linear models (LM) were employed to evaluate the overall linear trend of BMI and overweight rate across admission years. Additionally, logistic regression models were utilized to estimate the annual trend in overweight risk and the temporal trajectories of overweight prevalence. To capture potential non-linear trajectories, generalized additive models (GAM) were implemented using non-parametric smoothing techniques. Smoothing terms were fitted using penalized regression splines implemented in the mgcv package, with the basis dimension specified as *k* = 5. Model fit was evaluated based on the effective degrees of freedom (edf) and deviance explained reported by the fitted models. Additionally, another GAM was fitted using only observed data from 2016 to 2020 and 2022–2024. Because no freshmen were enrolled in 2021 owing to COVID-19-related disruptions, no observations were available for that year. To avoid introducing potential smoothing artifacts associated with model-based imputation, the missing year was not estimated and was excluded from the GAM fitting process.

The temporal impact of COVID-19 was analyzed by categorizing the study period into three distinct phases: pre-pandemic (2016–2019), pandemic (2020–2022), and post-pandemic (2023–2024). This classification was informed by policy stringency trends reported by the Oxford COVID-19 Government Response Tracker and the nationwide relaxation of China’s COVID-19 control measures at the end of 2022 (14). To evaluate the impact of COVID-19, a three-phase Interrupted Time Series (ITS) model was constructed. The model evaluated several key parameters: the baseline trend (*β*_1_), immediate level shifts (*β*_2_, *β*_3_) occurring at the onset of the pandemic and post-pandemic periods, and the subsequent changes in slopes (*β*_4_, *β*_5_) (19). Autocorrelation was performed to account for potential temporal dependencies between annual observations. Specifically, a first-order autoregressive correlation structure [corAR(1)] was integrated into generalized least squares models to adjust for serial correlation, thereby ensuring the statistical validity of the estimated *p*-values. Residual autocorrelation was assessed through model diagnostic procedures, and the AR(1) structure was retained when appropriate. To assess the potential influence of changing cohort composition, additional sensitivity analyses adjusting for sex and academic discipline were performed for the ITS models.


$$\begin{array}{l} Y_{t}=\beta_{0}+\beta_{1}{Τ}_{t}+\beta_{2}{X_{t}}^{\left(1\right)}+\beta_{3}{X_{t}}^{\left(2\right)}+\beta_{4}({Τ}_{t}-t_{1}){X_{t}}^{\left(1\right)}\\ +\beta_{5}({Τ}_{t}-t_{2}){X_{t}}^{\left(2\right)}+\varepsilon_{t} \end{array}$$

To investigate potential disparities in BMI and overweight trajectories across different subgroups, analyses were repeated based on sex (male vs. female) (22) and major STEM vs. HASS. The specific classification of majors into these two categories is presented in Supplementary Table 1. Subgroup comparisons were conducted using stratified ITS models to evaluate disparities between sexes (male vs. female) and academic disciplines (STEM vs. HASS).

All analyses were processed in R 4.3.0 (R Foundation for Statistical Computing, Vienna, Austria). Data cleaning and analysis were conducted using the dplyr, tidyr, and data.table packages, while graphical visualizations were generated using ggplot2. A GAM was implemented using the mgcv package. ITS analyses were conducted using the nlme package for generalized least squares models with a first-order autoregressive correlation structure (corAR1), whereas binomial generalized linear models were fitted using the baseline stats package. All statistical tests were two-sided, and a level of *p* < 0.05 was considered statistically significant.

## Results

A total of 39,478freshmen were included from 2016 to 2024, with annual sample sizes ranging from 4,469 to 5,243. Table 1 presents the demographic characteristics and BMI distribution of the study participants. The mean age of participants remained relatively stable across years (approximately 18.4–18.7 years). Female students consistently accounted for around two-thirds of the sample (63.1–66.2%), and the proportion of students majoring in STEM fields increased slightly over time, from 63.8% in 2016 to approximately 66.2–70.7% in later years.

**Table 1:** Characteristics and body mass index profile of the study participants.

Year	Sample size (*N*)	Age, mean ± SD	Sex, *n* (%) female	Major, % STEM	BMI, mean ± SD	Overweight rate, % (*n*/*N*) (BMI ≧ 25 kg/m^2^)	Overweight rate, % (*n*/*N*) (BMI ≧ 24 kg/m^2^)
2016	4,469	18.73 ± 0.91	2,819 (63.1%)	63.80%	21.03 ± 3.09	9.6% (427/4469)	14.1% (632/4469)
2017	4,577	18.56 ± 0.86	2,961 (64.7%)	66.20%	21.19 ± 3.38	11.9% (546/4577)	16.7% (764/4577)
2018	4,845	18.47 ± 0.78	3,189 (65.8%)	68.20%	21.21 ± 3.24	11.3% (548/4845)	16.1% (780/4845)
2019	5,115	18.48 ± 0.78	3,339 (65.3%)	70.70%	21.39 ± 3.67	13.8% (708/5115)	18.8% (962/5115)
2020	5,243	18.47 ± 0.78	3,357 (64.0%)	70.10%	21.52 ± 3.39	13.8% (722/5243)	19.2% (1,006/5243)
2022	5,091	18.37 ± 0.74	3,368 (66.2%)	70.10%	21.27 ± 3.32	12.2% (623/5091)	17.7% (900/5091)
2023	5,035	18.43 ± 0.70	3,300 (65.5%)	70.20%	21.56 ± 3.51	14.9% (752/5035)	20.7% (1,041/5035)
2024	5,103	18.47 ± 0.73	3,352 (65.7%)	69.60%	22.08 ± 4.82	20.6% (1,053/5103)	26.7% (1,364/5103)

SD, standard deviation; STEM, science, technology, engineering, and mathematics; BMI, body mass index.

The mean BMI showed an increasing trend during the study period, rising from 21.03 ± 3.09 kg/m^2^ in 2016 to 22.08 ± 4.82 kg/m^2^ in 2024. Between 2016 and 2022, the prevalence of overweight fluctuated between 9.6 and 12.2%, but increased markedly to 20.6% in 2024.

**Figure 2:**
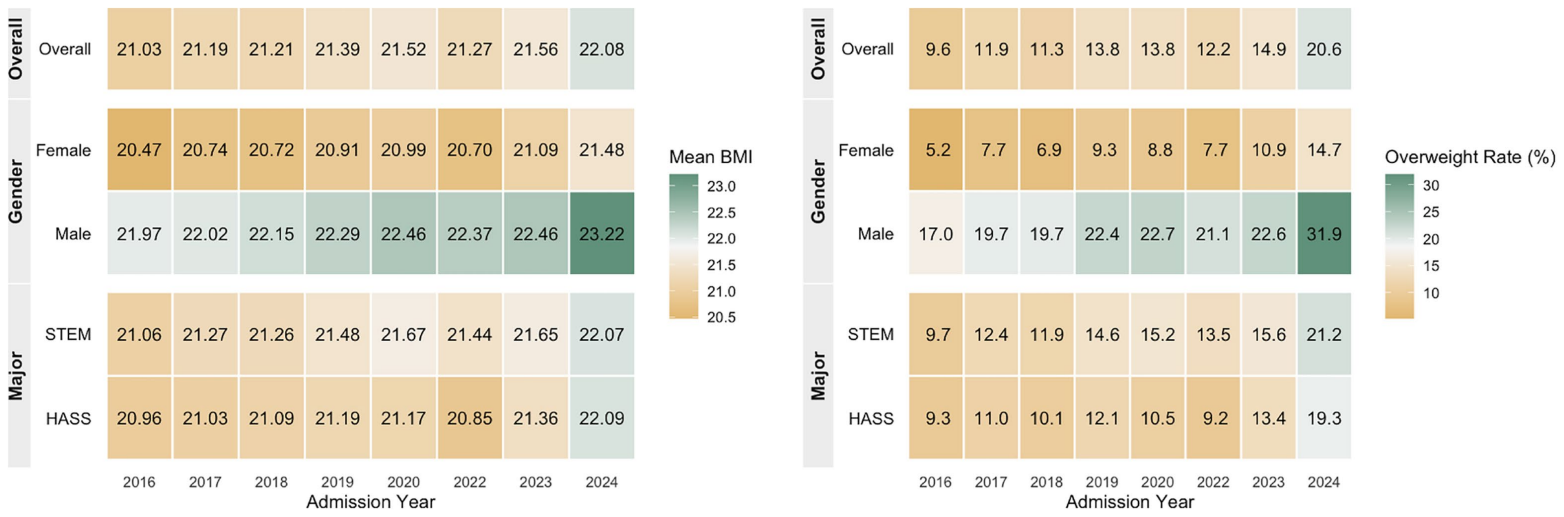
The dynamic evolution of mean body mass index and temporal trends in overweight rate among the study participants. BMI, body mass index.

Figure 2 illustrates the dynamic evolution of mean BMI and overweight rate from 2016 to 2024. Overall, the overweight rate and BMI showed a gradual increase over time. Male participants exhibited persistently higher mean BMI and overweight rate than female participants throughout the study period, with a more pronounced increase in overweight rate. When stratified by academic major, similar temporal patterns were observed among STEM and HASS participants.

To assess the overall temporal trends of BMI and overweight rate from 2016 to 2024, linear regression and GAMs were employed (Supplementary Table 2). Linear regression analyses revealed a steady and significant increasing trend in both mean BMI (*β* = 0.09, 95% CI 0.08–0.11, *p* < 0.001; Supplementary Figure 1) and overweight prevalence (*β* = 0.009, 95% CI 0.008–0.011, *p* < 0.001; Supplementary Figure 2). Specifically, the BMI trend exhibited a notable plateau period between 2020 and 2022, followed by an accelerated increase post-2022 (Supplementary Figure 3). Similarly, the logistic GAM confirmed a highly significant non-linear increase in overweight risk (*p* < 0.001; Supplementary Figure 4).

**Figure 3:**
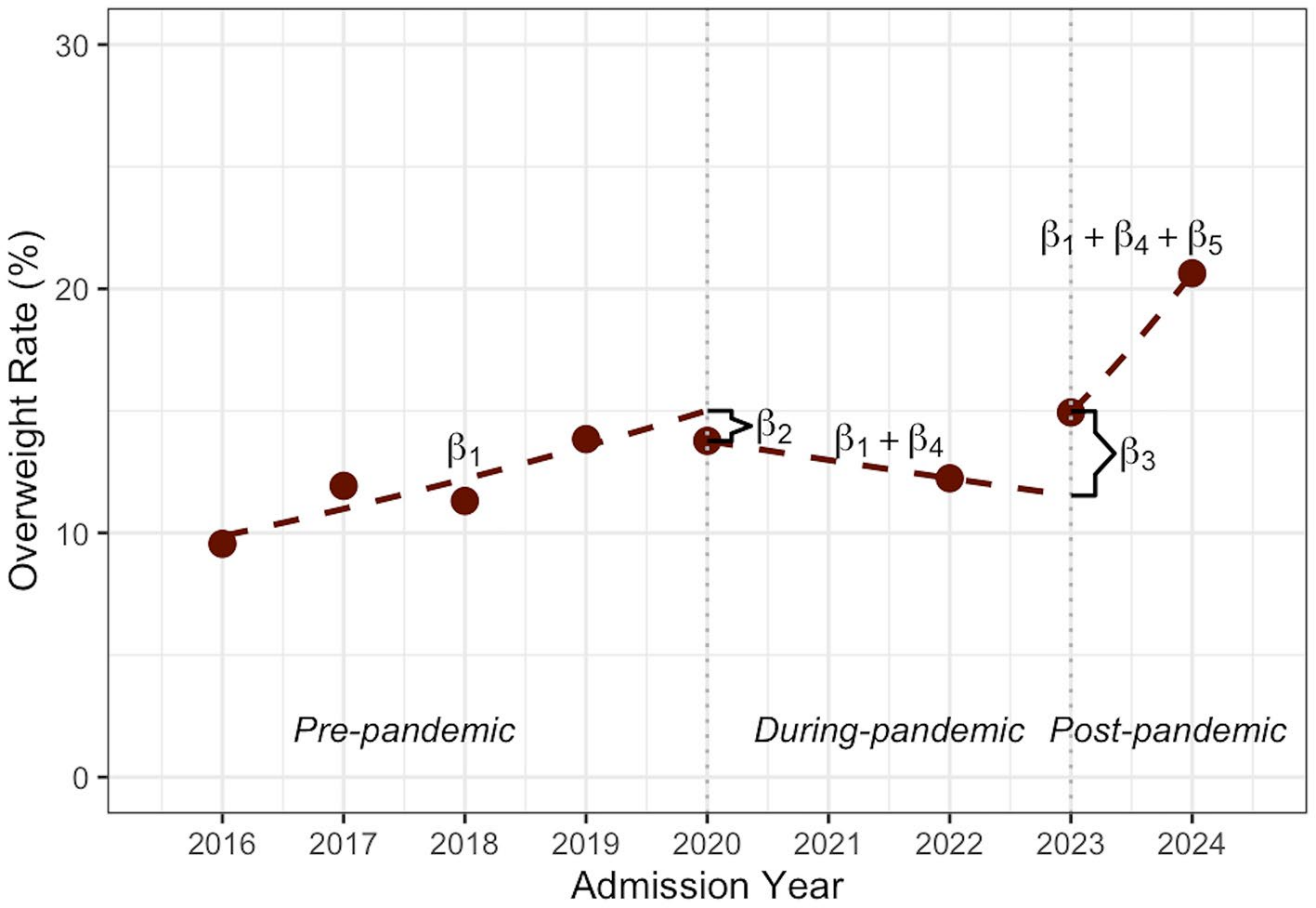
Interrupted time series analysis of overweight rate trends before, during, and after COVID-19. Parameters *β*1 through *β*5 represent the estimated effects from the Interrupted Time Series (ITS) model:·*β*1 (Baseline Trend): The annual change in BMI/Overweight rate during the pre-pandemic period (2016–2019).·*β*2 (Step Change 2020): The immediate level shift in the outcome at the onset of the COVID-19 pandemic (2020). *β*3 (Step Change 2023): The immediate level shift in the outcome when the “Zero-COVID” policy ended (2023). *β*4 (Trend Change 1): The change in the annual slope during the pandemic period compared to the pre-pandemic trend. *β*5 (Trend Change 2): The change in the annual slope during the post-pandemic period compared to the during-pandemic trend.

The ITS analysis revealed distinct temporal shifts in students’ BMI and overweight rate (Table 2). During the pre-pandemic period (2016–2019), both BMI and overweight prevalence showed significant increasing trends (BMI: *β*_1_ = 0.096, *p* < 0.001; overweight: *β*_1_ = 0.120, *p* < 0.001). At the onset of COVID-19 in 2020, a significant immediate increase was observed in mean BMI (*β*_2_ = 0.125, *p* = 0.008). However, the immediate change in overweight prevalence was not statistically significant. This pattern suggests that changes in BMI may have occurred earlier than changes in overweight prevalence, as increases in body weight may not immediately result in a sufficient proportion of students exceeding the overweight threshold. During the pandemic period (2020–2022), the growth trends of both BMI and overweight slowed significantly (BMI: *β*_4_ = −0.222, *p* < 0.001; overweight: *β*_4_ = −0.187, *p* < 0.001). After the termination of the “Zero-COVID” policy in 2023, both BMI and overweight rates showed significantly level increases (BMI: *β*_3_ = 0.327, *p* = 0.007; overweight: *β*_3_ = 0.298, *p* < 0.001) and accelerated growth trends (BMI: *β*_5_ = 0.738, *p* = 0.001; overweight: *β*_5_ = 0.460, *p* < 0.001), contributing to the marked increases observed by 2024 (Figure 3 and Supplementary Figure 5). As a sensitivity analysis, all overweight-related analyses were repeated using the WGOC criterion (BMI ≥ 24 kg/m^2^). Although the lower cutoff increased the absolute prevalence of overweight across all study years, the overall temporal patterns remained highly consistent with the primary analysis based on the WHO definition (BMI ≥ 25 kg/m^2^) (Supplementary Tables 3, 4 and Supplementary Figures 6, 7).

**Table 2:** Parameter estimates in models for freshmen body mass index and overweight rate: an interrupted time series analysis.

Parameters	BMI			Overweight (BMI ≧ 25 kg/m^2^)			Overweight (BMI ≧ 24 kg/m^2^)		
	*β*	SE	*p*-value	*β*	SE	*p*-value	*β*	SE	*p*-value
*β*_1_	0.096	0.002	<0.001	0.120	0.020	<0.001	0.098	0.018	<0.001
*β*_2_	0.125	0.011	0.008	−0.101	0.066	0.125	−0.053	0.058	0.362
*β*_3_	0.327	0.027	0.007	0.298	0.078	<0.001	0.244	0.068	<0.001
*β*_4_	−0.222	0.005	<0.001	−0.187	0.036	<0.001	−0.148	0.031	<0.001
*β*_5_	0.738	0.024	0.001	0.460	0.060	<0.001	0.386	0.053	<0.001

BMI, body mass index; SE, standard error.

Parameters *β*1 through β5 represent the estimated effects from the interrupted time series (ITS) model.

*β*1 (Baseline Trend): The annual change in BMI/Overweight rate during the pre-pandemic period (2016–2019).

*β*2 (Step Change 2020): The immediate level shift in the outcome at the onset of the COVID-19 pandemic (2020).

*β*3 (Step Change 2023): The immediate level shift in the outcome when the “Zero-COVID” policy ended (2023).

*β*4 (Trend Change 1): The change in the annual slope during the pandemic period compared to the pre-pandemic trend.

*β*5 (Trend Change 2): The change in the annual slope during the post-pandemic period compared to the during-pandemic trend.

Stratified ITS analyses confirmed that these three-phase trajectories were broadly consistent across the overall participants and academic subgroups. Sex-stratified analyses further revealed clear gender differences (Supplementary Figures 8, 9). Male students consistently had higher mean BMI and overweight prevalence than female students throughout the study period. After the termination of the pandemic control policies in 2023, both sexes showed an increasing trend. However, males experienced a more pronounced rebound, with overweight prevalence surpassing 30% in 2024, accompanied by steeper increases in both the level change (*β*_3_) and slope change (*β*_5_). In contrast, females also exhibited a significant level increase in 2023 (*β*_3_), but the overall magnitude of change was smaller, with overweight prevalence remaining below 15%.

Similarly, discipline-stratified analysis (Supplementary Figures 10, 11) showed consistent differences between academic groups. Students in STEM majors consistently exhibited higher mean BMI and overweight prevalence than those in HASS majors. Following the termination of pandemic control policies in 2023, both groups experienced a rebound in BMI and overweight prevalence. However, the post-pandemic increase (*β*_1_ + *β*_4_ + *β*_5_) was more pronounced among the STEM students. As a result, the gap between the two groups widened in the post-pandemic period. By 2024, overweight prevalence among the STEM group approached 30%, a level substantially higher than that observed in the HASS group.

## Discussion

Overall, this study indicates that mean BMI and overweight prevalence among university students increased over the study period. BMI trajectories were non-linear over time, characterized by an increasing in the pre-pandemic period, and a pronounced increase during the post-pandemic period. Subgroup analyses revealed heterogeneity, with steeper increases observed in males. By discipline, HASS students showed a greater immediate post-pandemic increase, whereas STEM students exhibited a more sustained upward trend over time.

The increasing trend in BMI prior to the pandemic likely reflects the transition from a structured pre-university environment to a more autonomous university setting (10, 23, 24) which is associated with greater behavioral flexibility and increased exposure to obesogenic environments. This interpretation is supported by the progressively higher BMI observed at university entry across successive cohorts, suggesting a cumulative influence of pre-university environmental and behavioral factors. During the pandemic, mobility restrictions, reduced social interaction, and decreased exposure to external food environments may have temporarily constrained obesogenic exposures (25, 26). However, this phase may reflect a temporary suppression of risk behaviors rather than a sustained improvement in health status. Importantly, these effects may have been particularly evident in China, where the “Zero-COVID” strategy involved relatively stringent and prolonged restrictions (27). Notably, 2022 likely represented the peak intensity of these control measures, characterized by continued mobility restrictions and extensive public health interventions (14). The pronounced increase observed in 2023–2024 may partly reflect an “opening-up effect” following the abrupt termination of China’s “Zero-COVID” policy. After prolonged restrictions on movement and social interaction, the rapid return to normal life may have generated a rebound in social and consumption behaviors, including more frequent dining out and social gatherings (28). Such changes may have increased exposure to obesogenic environments and contributed to the accelerated rise in BMI and overweight prevalence during the post-pandemic period.

Sex-specific patterns further suggest differential responses to these environmental and behavioral changes. Across all phases, male students consistently exhibited higher BMI levels and a greater prevalence of overweight compared to females, along with a more pronounced increase in the post-pandemic period. This pattern may reflect a higher baseline susceptibility to obesogenic exposures among males, potentially related to differences in dietary behaviors, physical activity patterns, and health awareness (28). In contrast, female students showed relatively lower BMI levels and a more moderate increase over time, which may be associated with stronger weight-control behaviors and greater sensitivity to body image norms (29). Notably, the post-pandemic acceleration appeared more pronounced among males, suggesting that the combined effects of behavioral rebound and persistent sedentary habits may have disproportionately affected this group.

Discipline-specific differences further highlight distinct underlying mechanisms. HASS students appeared more responsive to immediate environmental changes, exhibiting a more pronounced short-term increase following transitions. In contrast, STEM students showed a more sustained upward trajectory, potentially reflecting the cumulative effects of prolonged sedentary study patterns and academic workload. In addition, academic stress may represent another potential mechanism underlying the sustained increase observed among STEM students. Previous studies have shown that academic stress is associated with unhealthy dietary behaviors, sleep disruption, reduced physical activity, and greater weight gain among university students (30, 31). The combined effects of these behavioral responses may contribute to long-term increases in BMI and overweight prevalence.

Previous studies have shown that the COVID-19 pandemic is associated with reduced physical activity, increased sedentary behavior, and weight gain across diverse populations (15, 16, 32). The findings of the present study are broadly consistent with existing literature, suggesting that similar trends have been observed in previous research. However, most prior studies have primarily focused on short-term changes during the pandemic period, with limited attention to longer-term trajectories following the lifting of restrictions (10). By incorporating data spanning the pre-, during-, and post-pandemic periods, this study extends existing evidence by elucidating the temporal evolution of these trends. Specifically, the observed post-pandemic increase may reflect a continued and potentially accelerated trajectory rather than a simple reversion to pre-pandemic levels. Furthermore, while earlier research has often treated university students as a relatively homogeneous population (33), the identification of discipline-specific differences in this study highlights the importance of accounting for subgroup heterogeneity in the assessment of health trajectories.

The observed increase in BMI during the post-pandemic period underscores the need for effective and scalable interventions within university settings. These findings suggest that health promotion strategies should move beyond a one-size-fits-all approach and instead adopt more targeted interventions. From a gender perspective, male students exhibited a higher prevalence of overweight and a more rapid increase over time, highlighting the importance of gender-sensitive strategies, particularly those aimed at promoting physical activity and facilitating early risk identification (34). In addition, discipline-specific approaches are necessary to address different behavioral patterns. For HASS students, interventions may focus on mitigating socially driven eating behaviors, whereas for STEM students, efforts should prioritize reducing prolonged sedentary time and preventing the accumulation of long-term behavioral risks. To enhance the long-term sustainability, these interventions should be integrated into university systems, including structured physical activity programs, improved nutrition labeling in campus dining environments, and the integration of digital health monitoring tools (35). Such approaches may support a transition from short-term responses to more sustained health promotion in the post-pandemic context.

While this study provides important insights into BMI trajectories among university students across the pre-, during-, and post-pandemic periods, several limitations should be considered when interpreting the findings. First, the study was conducted within a single university, which may limit the generalizability of the results to populations in other regions. Local dietary environments, campus management policies, and student demographic characteristics may differ substantially across institutions and geographic areas, potentially influencing BMI and overweight trajectories. Second, although the repeated cross-sectional design is appropriate for assessing population-level trends over time, it does not allow for tracking individual-level changes or establishing causal relationships. Therefore, the observed ITS effects should be interpreted as aggregate population-level shifts rather than individual weight gain trajectories. Third, the post-pandemic period included only 2 years of observations (2023–2024). Consequently, the estimated level and slope changes in the ITS analyses should be interpreted with caution, as they may be sensitive to short-term fluctuations. In addition, as data for 2021 were unavailable due to the pandemic, the temporal continuity of the series was disrupted. The limited number of post-pandemic observations restricts the precision of estimates regarding post-pandemic trends. Longer follow-up will be needed to confirm the persistence and magnitude of these changes. In addition, the absence of detailed behavioral data, such as dietary patterns, sleep, and physical activity, precludes the ability to fully explain the underlying mechanisms of BMI change. Future research should extend these findings by conducting multi-center studies and incorporating objective measurement tools, such as wearable devices, to enable longitudinal tracking of individuals and better capture dynamic behavioral changes over time.

## Conclusion

This study demonstrates that BMI and overweight prevalence among university students increased over the study period, with the post-pandemic increase substantially exceeding the pre-pandemic trend. The findings further reveal widening disparities across subgroups, as male students and those in STEM disciplines experienced more pronounced post-pandemic increases in BMI and overweight prevalence. These results suggest that the lifting of COVID-19 restrictions may have accelerated existing upward trends rather than simply restoring pre-pandemic patterns. Given the growing burden of overweight and the emergence of subgroup-specific vulnerabilities, targeted public health interventions such as weight management and health promotion programs should be prioritized, particularly for high-risk groups such as male and STEM students. Future longitudinal cohort studies are needed to clarify causal relationships and underlying mechanisms, particularly through tracking individual behavioral changes over time.

## Data Availability

The data analyzed in this study is subject to the following licenses/restrictions: Data will be available upon reasonable request from the corresponding authors. Requests to access these datasets should be directed to JJ, jianguangji@um.edu.mo; ZZ, zhangzhifeng@hebeinu.edu.cn.

## References

[1] World Health Organization. Obesity and Overweight. World Health Organization (2025). Available online at: https://www.who.int/news-room/fact-sheets/detail/obesity-and-overweight (Accessed March 13, 2026)

[2] NCD Risk Factor Collaboration (NCD-RisC). Worldwide trends in underweight and obesity from 1990 to 2022: a pooled analysis of 3663 population-representative studies with 222 million children, adolescents, and adults. Lancet. (2024). 403:1027–50. doi: 10.1016/S0140-6736(23)02750-2, 38432237 PMC7615769

[3] Powell-Wiley TM Poirier P Burke LE Després JP Gordon-Larsen P Lavie CJ . Obesity and cardiovascular disease: a scientific statement from the American Heart Association. Circulation. (2021) 143:e984–e1010. doi: 10.1161/CIR.0000000000000973, 33882682 PMC8493650

[4] Lauby-Secretan B Scoccianti C Loomis D Grosse Y Bianchini F Straif K . Body fatness and Cancer--viewpoint of the IARC working group. N Engl J Med. (2016) 375:794–8. doi: 10.1056/NEJMsr1606602, 27557308 PMC6754861

[5] GBD 2021 Risk Factors Collaborators. Global burden and strength of evidence for 88 risk factors in 204 countries and 811 subnational locations, 1990-2021: a systematic analysis for the global burden of disease study 2021. Lancet. (2024). 403:2162–203. doi: 10.1016/S0140-6736(24)00933-4, 38762324 PMC11120204

[6] Zhao S Xu X You H Ge J Wu Q. Healthcare costs attributable to abnormal weight in China: evidence based on a longitudinal study. BMC Public Health. (2023) 23:1927. doi: 10.1186/s12889-023-16855-6, 37798694 PMC10552200

[7] Qin X Pan J. The medical cost attributable to obesity and overweight in China: estimation based on longitudinal surveys. Health Econ. (2016) 25:1291–311. doi: 10.1002/hec.3217 26223895

[8] Tsur AM Akavian I Landau R Derazne E Tzur D Vivante A . Adolescent body mass index and early chronic kidney disease in young adulthood. JAMA Pediatr. (2024) 178:142–50. doi: 10.1001/jamapediatrics.2023.5420, 38079159 PMC10714283

[9] Ruiz LD Zuelch ML Dimitratos SM Scherr RE. Adolescent obesity: diet quality, psychosocial health, and cardiometabolic risk factors. Nutrients. (2019) 12:43. doi: 10.3390/nu12010043, 31877943 PMC7020092

[10] Vadeboncoeur C Townsend N Foster C. A meta-analysis of weight gain in first year university students: is freshman 15 a myth? BMC Obes. (2015) 2:22. doi: 10.1186/s40608-015-0051-7, 26217537 PMC4511069

[11] Twig G Yaniv G Levine H Leiba A Goldberger N Derazne E . Body-mass index in 2.3 million adolescents and cardiovascular death in adulthood. N Engl J Med. (2016) 374:2430–40. doi: 10.1056/NEJMoa1503840, 27074389

[12] Dun Y Ripley-Gonzalez JW Zhou N You B Li Q Li H . Weight gain in Chinese youth during a 4-month COVID-19 lockdown: a retrospective observational study. BMJ Open. (2021) 11:e052451. doi: 10.1136/bmjopen-2021-052451, 34301671 PMC8300557

[13] Cheng ZJ Zhan Z Xue M Zheng P Lyu J Ma J . Public health measures and the control of COVID-19 in China. Clin Rev Allergy Immunol. (2023) 64:1–16. doi: 10.1007/s12016-021-08900-2, 34536214 PMC8449219

[14] Hale T Angrist N Goldszmidt R Kira B Petherick A Phillips T . A global panel database of pandemic policies (Oxford COVID-19 government response tracker). Nat Hum Behav. (2021) 5:529–38. doi: 10.1038/s41562-021-01079-8, 33686204

[15] Stockwell S Trott M Tully M Shin J Barnett Y Butler L . Changes in physical activity and sedentary behaviours from before to during the COVID-19 pandemic lockdown: a systematic review. BMJ Open Sport Exerc Med. (2021) 7:e000960. doi: 10.1136/bmjsem-2020-000960, 34192010 PMC7852071

[16] Ammar A Brach M Trabelsi K Chtourou H Boukhris O Masmoudi L . Effects of COVID-19 home confinement on eating behaviour and physical activity: results of the ECLB-COVID19 international online survey. Nutrients. (2020) 12:1583. doi: 10.3390/nu12061583, 32481594 PMC7352706

[17] Plotnikoff RC Costigan SA Williams RL Hutchesson MJ Kennedy SG Robards SL . Effectiveness of interventions targeting physical activity, nutrition and healthy weight for university and college students: a systematic review and meta-analysis. Int J Behav Nutr Phys Act. (2015) 12:45. doi: 10.1186/s12966-015-0203-7, 25890337 PMC4393577

[18] Ma J Cai CH Wang HJ Dong B Song Y Hu PJ . The trend analysis of overweight and obesity in Chinese students during 1985–2010. Zhonghua Yu Fang Yi Xue Za Zhi. (1985) 46:776–80. 23157879

[19] Zhang B Liu W Lemon SC Barton BA Fischer MA Lawrence C . Design, analysis, power, and sample size calculation for three-phase interrupted time series analysis in evaluation of health policy interventions. J Eval Clin Pract. (2020) 26:826–41. doi: 10.1111/jep.13266, 31429175 PMC7028460

[20] Zhou B Coorperative Meta-Analysis Group of China Obesity Task Force. Predictive values of body mass index and waist circumference to risk factors of related diseases in Chinese adult population. Zhonghua Liu Xing Bing Xue Za Zhi. (2002) 23:5–10. 12015100

[21] UNESCO Institute for Statistics. International Standard Classification of Education: Fields of Education and Training 2013 (ISCED-F 2013). Montreal: UNESCO Institute for Statistics (2015).

[22] Mäkinen VP Ala-Korpela M. Influence of age and sex on longitudinal metabolic profiles and body weight trajectories in the UK biobank. Int J Epidemiol. (2024) 53:dyae055. doi: 10.1093/ije/dyae055, 38641429 PMC11031410

[23] Levitsky DA Halbmaier CA Mrdjenovic G. The freshman weight gain: a model for the study of the epidemic of obesity. Int J Obes Relat Metab Disord. (2004) 28:1435–42. doi: 10.1038/sj.ijo.0802776, 15365585

[24] Boyce JA Kuijer RG. Perceived stress and freshman weight change: the moderating role of baseline body mass index. Physiol Behav. (2015) 139:491–6. doi: 10.1016/j.physbeh.2014.12.011 25484356

[25] Rivera PA Nys BL Fiestas F. Impact of COVID-19 induced lockdown on physical activity and sedentary behavior among university students: a systematic review. Medwave. (2021) 21:e8456–6. doi: 10.5867/medwave.2021.08.8456 34487515

[26] Di Renzo L Gualtieri P Pivari F Soldati L Attinà A Cinelli G . Eating habits and lifestyle changes during COVID-19 lockdown: an Italian survey. J Transl Med. (2020) 18:229. doi: 10.1186/s12967-020-02399-5, 32513197 PMC7278251

[27] Jia J.S. Lu X. Yuan Y. Xu G. Jia J. Christakis N. A. Population flow drives spatio-temporal distribution of COVID-19 in China Nature 582 389–394 (2020) doi: 10.1038/s41586-020-2284-y 32349120

[28] International Monetary Fund (2023) People’s Republic of China: 2023 Article IV Consultation Mission Washington, DC. Available online at: https://www.imf.org/en/news/articles/2023/11/07/pr23380-imf-staff-completes-2023-article-iv-mission-to-the-peoples-republic-of-china (Accessed June 5, 2026)

[29] Feraco A Armani A Gorini S Camajani E Quattrini C Filardi T . Gender differences in dietary patterns and eating behaviours in individuals with obesity. Nutrients. (2024) 16:4226. doi: 10.3390/nu16234226, 39683619 PMC12121239

[30] Lyzwinski LN Caffery L Bambling M Edirippulige S. The relationship between stress and maladaptive weight-related behaviors in college students: a review of the literature. Am J Health Educ. (2018) 49:166–78. doi: 10.1080/19325037.2018.1449683

[31] Wunsch K Fiedler J Bachert P Woll A. The tridirectional relationship among physical activity, stress, and academic performance in university students: a systematic review and meta-analysis. Int J Environ Res Public Health. (2021) 18:739. doi: 10.3390/ijerph18020739, 33467118 PMC7830011

[32] Bakaloudi DR Barazzoni R Bischoff SC Breda J Wickramasinghe K Chourdakis M. Impact of the first COVID-19 lockdown on body weight: a combined systematic review and a meta-analysis. Clin Nutr. (2022) 41:3046–54. doi: 10.1016/j.clnu.2021.04.015, 34049749 PMC8056819

[33] El Ansari W Suominen S Samara A. Eating habits and dietary intake: is adherence to dietary guidelines associated with importance of healthy eating among undergraduate university students in Finland? Cent Eur J Public Health. (2015) 23:306–13. doi: 10.21101/cejph.a4195 26841143

[34] Guthold R Stevens GA Riley LM Bull FC. Worldwide trends in insufficient physical activity from 2001 to 2016: a pooled analysis of 358 population-based surveys with 1·9 million participants. Lancet Glob Health. (2018) 6(10):e1077–e1086. doi: 10.1016/S2214-109X(18)30357-7. Erratum in: Lancet Glob Health. 2019 7: e36. doi: 10.1016/S2214-109X(18)30454-6. 30193830, . 30193830

[35] Story M Kaphingst KM Robinson-O'Brien R Glanz K. Creating healthy food and eating environments: policy and environmental approaches. Annu Rev Public Health. (2008) 29:253–72. doi: 10.1146/annurev.publhealth.29.020907.090926, 18031223

