# Supplementary material for: Trends in overweight prevalence among university freshmen and the impact of the COVID-19 pandemic: a repeated cross-sectional study
Source: Front Public Health. 2026 Jul 1;14:1856579. doi: 10.3389/fpubh.2026.1856579 (PMC13368738; doi:10.3389/fpubh.2026.1856579)
Supplement: Supplementary file 1 [file Data_Sheet_1.docx]

Supplementary Material

**List of supplementary tables and figures**

**Supplementary Table 1.** Categorization of majors (HASS vs. STEM)

**Supplementary Table 2.** Body mass index and overweight trends

**Supplementary Table 3.** Parameter estimates in adjusted models for freshmen body mass index and overweight rate: an interrupted time series analysis

**Supplementary Table 4.** Subgroup analyses and interaction tests for temporal trends in BMI and overweight rate according to sex and academic discipline

**Supplementary Figure 1.** Linear trend of freshmen body mass index

**Supplementary Figure 2.** Linear trends in overweight rate among freshmen

**Supplementary Figure 3.** Nonlinear temporal trends in freshmen body mass index

**Supplementary Figure 4.** Nonlinear temporal trends in overweight rate

**Supplementary Figure 5.** Interrupted time series analysis of mean BMI trends before and after COVID-19

**Supplementary Figure 6.** The temporal trends in overweight rate among the study participants using BMI ≥ 24 kg/m²

**Supplementary Figure 7.** Interrupted time series analysis of overweight rate trends before, during, and after COVID-19 using BMI ≥ 24 kg/m²

**Supplementary Figure 8.** Interrupted time series analysis of mean BMI trends before, during, and after COVID-19 by sex

**Supplementary Figure 9.** Interrupted time series analysis of overweight rate trends before, during, and after COVID-19 by sex

**Supplementary Figure 10.** Interrupted time series analysis of mean BMI trends before, during, and after COVID-19 by discipline

**Supplementary Figure 11.** Interrupted time series analysis of overweight rate trends before, during, and after COVID-19 by discipline

**Supplementary Table 1.** Categorization of majors (HASS vs. STEM)

| **HASS** | | | | |
| --- | --- | --- | --- | --- |
| Hotel Management | Ideological and Political Education | Financial Management | Agriculture and Forestry Management | Human Resource Management |
| Marketing | Economics and Finance | Japanese | Business English | English |
| Teaching Chinese as a Foreign Language | Chinese Language and Literature | Secretarial Studies | Russian | Preschool Education |
| Applied Psychology | Social Sports Guidance | Physical Education | Theatre, Film and Television | Environmental Design |
| Visual Communication Design | Digital Media | Dance Performance | Dance Studies | Musicology |
| Fine Arts | History |  |  |  |
| **STEM** | | | | |
| Grassland Science | Animal Science | Veterinary Pharmacy | Veterinary Medicine | Animal and Plant Quarantine |
| Agronomy | Horticulture | Plant Protection | Plant Science | Seed Science |
| Facility Agriculture | Smart Agriculture | Food Science | Food Quality and Safety | Chemistry |
| Applied Chemistry | New Energy Science and Engineering | New Energy | Energy Storage Science | Mathematics |
| Statistics | Information and Computing Science | Data Science | Physics | Electronic Engineering |
| Computer Science | Information Science | Medical Information Engineering | Electronic and Information Engineering | Electronic Information Science |
| Communication Engineering | Information Engineering | Intelligent Science | Nursing | Pharmaceutics |
| Pharmacy | Health Inspection | Medical Laboratory Science | Rehabilitation Therapy | Chinese Materia Medica |
| Acueuncture-Mexibustien and Tuina | Chinese-Western Integrative Medicine | Traditional Chinese Medicine | Forensic Medicine | Stomatology |
| Clinical Medicine | Anesthesiology | Medical Imaging | Preventive Medicine | Pharmaceutical Engineering |
| Bioinformatics | Biological Information |  |  |  |

Abbreviations: HASS, humanities, arts and social sciences; STEM, science, technology, engineering, and mathematics.

**Supplementary Table 2.** Body mass index and overweight trends

| **Variable** | | **BMI** | | | **Overweight**  **(BMI≧25 kg/m²)** | | | **Overweight**  **(BMI≧24 kg/m²)** | | |
| --- | --- | --- | --- | --- | --- | --- | --- | --- | --- | --- |
|  |  | **β** | **95% CI** | **P value** | **β** | **95% CI** | **P-value** | **β** | **95% CI** | **P-value** |
| Age | | 0.09 | 0.08 – 0.11 | <0.001 | 0.08 | 0.07 – 0.09 | <0.001 | 0.07 | 0.06 – 0.08 | <0.001 |
| Sex | Male | 0.12 | 0.09 – 0.14 | <0.001 | 0.07 | 0.06 – 0.08 | <0.001 | 0.06 | 0.05 – 0.07 | <0.001 |
|  | Female | 0.09 | 0.07 – 0.10 | <0.001 | 0.1 | 0.09 – 0.12 | <0.001 | 0.1 | 0.08 – 0.11 | <0.001 |
| Major | HASS | 0.09 | 0.06 – 0.11 | <0.001 | 0.07 | 0.05 – 0.09 | <0.001 | 0.07 | 0.05 – 0.09 | <0.001 |
|  | STEM | 0.09 | 0.08 – 0.11 | <0.001 | 0.08 | 0.07 – 0.09 | <0.001 | 0.07 | 0.06 – 0.08 | <0.001 |

Abbreviations: BMI, body mass index; HASS, humanities, arts and social sciences; STEM, science, technology, engineering, and mathematics.

**Supplementary Table 3.** Parameter estimates in adjusted models for freshmen body mass index and overweight rate: an interrupted time series analysis

| **Parameters** | **BMI (Adjusted)** | | | **Overweight**  **(BMI ≥ 25 kg/m²) (Adjusted)** | | | **Overweight**  **(BMI ≥ 24 kg/m²) (Adjusted)** | | |
| --- | --- | --- | --- | --- | --- | --- | --- | --- | --- |
|  | **β** | **SE** | **P-value** | **β** | **SE** | **P-value** | **β** | **SE** | **P-value** |
| β1 | 0.123 | 0.023 | < 0.001 | 0.131 | 0.021 | < 0.001 | 0.108 | 0.018 | < 0.001 |
| β2 | -0.002 | 0.078 | 0.977 | -0.132 | 0.067 | 0.048 | -0.080 | 0.059 | 0.174 |
| β3 | 0.395 | 0.093 | < 0.001 | 0.289 | 0.079 | < 0.001 | 0.236 | 0.069 | < 0.001 |
| β4 | -0.233 | 0.042 | < 0.001 | -0.189 | 0.036 | < 0.001 | -0.149 | 0.031 | < 0.001 |
| β5 | 0.631 | 0.078 | < 0.001 | 0.470 | 0.061 | < 0.001 | 0.394 | 0.055 | < 0.001 |

Abbreviations: BMI, body mass index; SE, standard error.

Notes: Parameters β1 through β5 represent the estimated effects from the Interrupted Time Series (ITS) model:

·β1 (Baseline Trend): The annual change in BMI/Overweight rate during the pre-pandemic period (2016–2019).

·β2 (Step Change 2020): The immediate level shift in the outcome at the onset of the COVID-19 pandemic (2020).

·β3 (Step Change 2023): The immediate level shift in the outcome when the “Zero-COVID” policy ended (2023).

·β4 (Trend Change 1): The change in the annual slope during the pandemic period compared to the pre-pandemic trend.

·β5 (Trend Change 2): The change in the annual slope during the post-pandemic period compared to the during-pandemic trend.

·For the adjusted model , all β parameters represent the estimated independent effects after adjusting for individual-level sex and academic discipline as covariates, with a random intercept for year to control for temporal autocorrelation.

**Supplementary Table 4.** Subgroup analyses and interaction tests for temporal trends in BMI and overweight rate according to sex and academic discipline

| **Subgroup Category** | | **Covariates** | **BMI** | | | **Overweight**  **(BMI ≥ 25 kg/m²) (Adjusted)** | | | **Overweight**  **(BMI ≥ 24 kg/m²) (Adjusted)** | | |
| --- | --- | --- | --- | --- | --- | --- | --- | --- | --- | --- | --- |
|  |  |  | **β** | **SE** | **Interaction P-value** | **OR** | **SE** | **Interaction P-value** | **OR** | **SE** | **Interaction P-value** |
| Sex | Male | Admission Year | 0.116 | 0.013 | 0.025 | 1.072 | 0.008 | < 0.001 | 1.062 | 0.007 | < 0.001 |
|  | Female | Admission Year | 0.085 | 0.007 |  | 1.108 | 0.008 |  | 1.100 | 0.007 |  |
| Discipline | STEM | Admission Year | 0.094 | 0.008 | 0.581 | 1.084 | 0.007 | 0.555 | 1.075 | 0.006 | 0.779 |
|  | HASS | Admission Year | 0.086 | 0.016 |  | 1.076 | 0.010 |  | 1.072 | 0.009 |  |

Notes: 1. β represents the regression coefficient from linear regression models for BMI.

2. OR (Odds Ratio) and SE (Standard Error) for Overweight were estimated using logistic regression models, where overweight was defined as a binary outcome

3. Interaction P-values were obtained from models including admission year × sex and admission year × academic discipline interaction terms and were used to assess whether temporal trends differed significantly between subgroups.

**Supplementary Figure 1.** Linear trend of freshmen body mass index

**
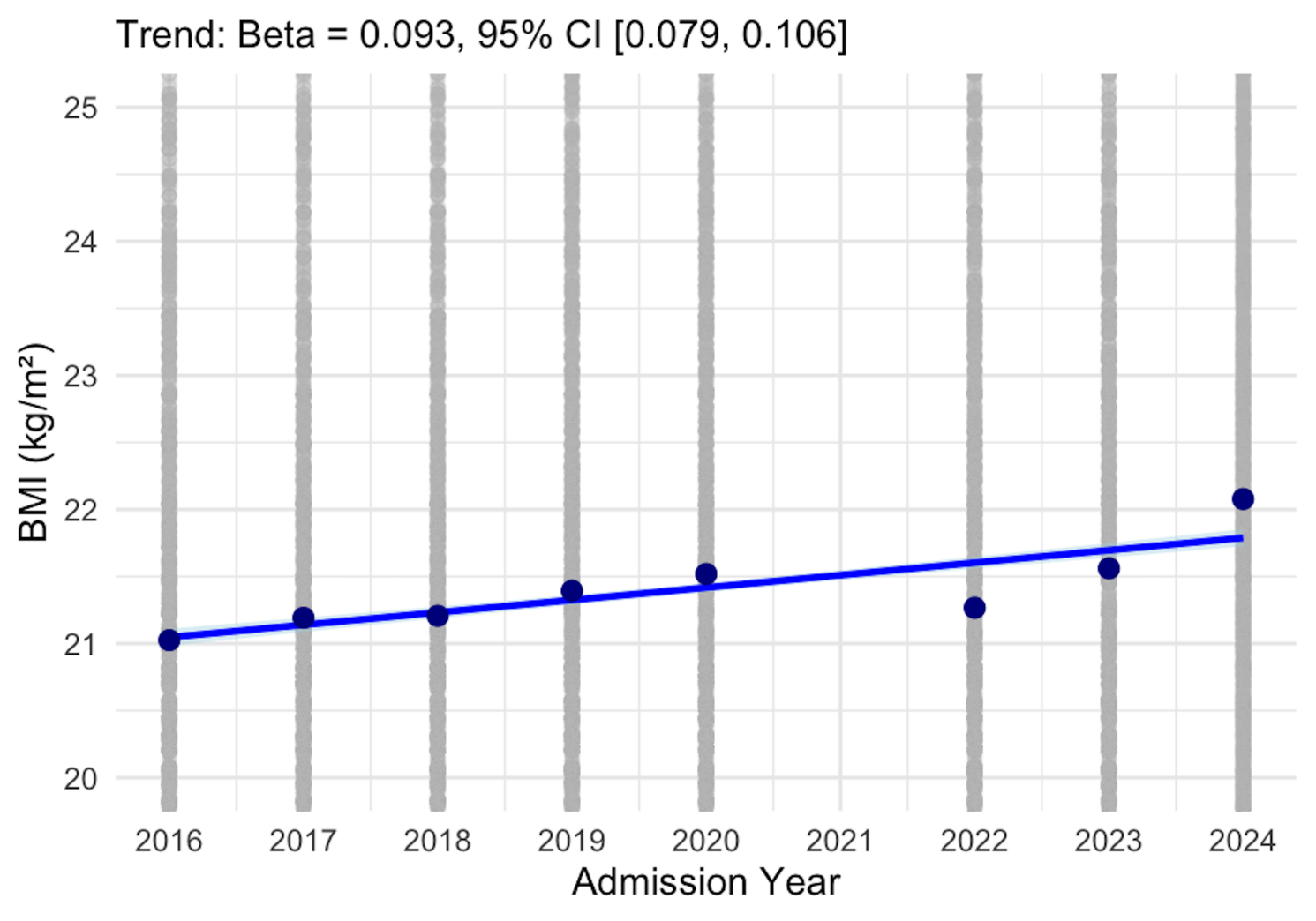
**

Abbreviations: BMI, body mass index; CI, confidence interval.

**Supplementary Figure 2.** Linear trends in overweight rate among freshmen

(A) BMI ≥ 25 kg/m²

**
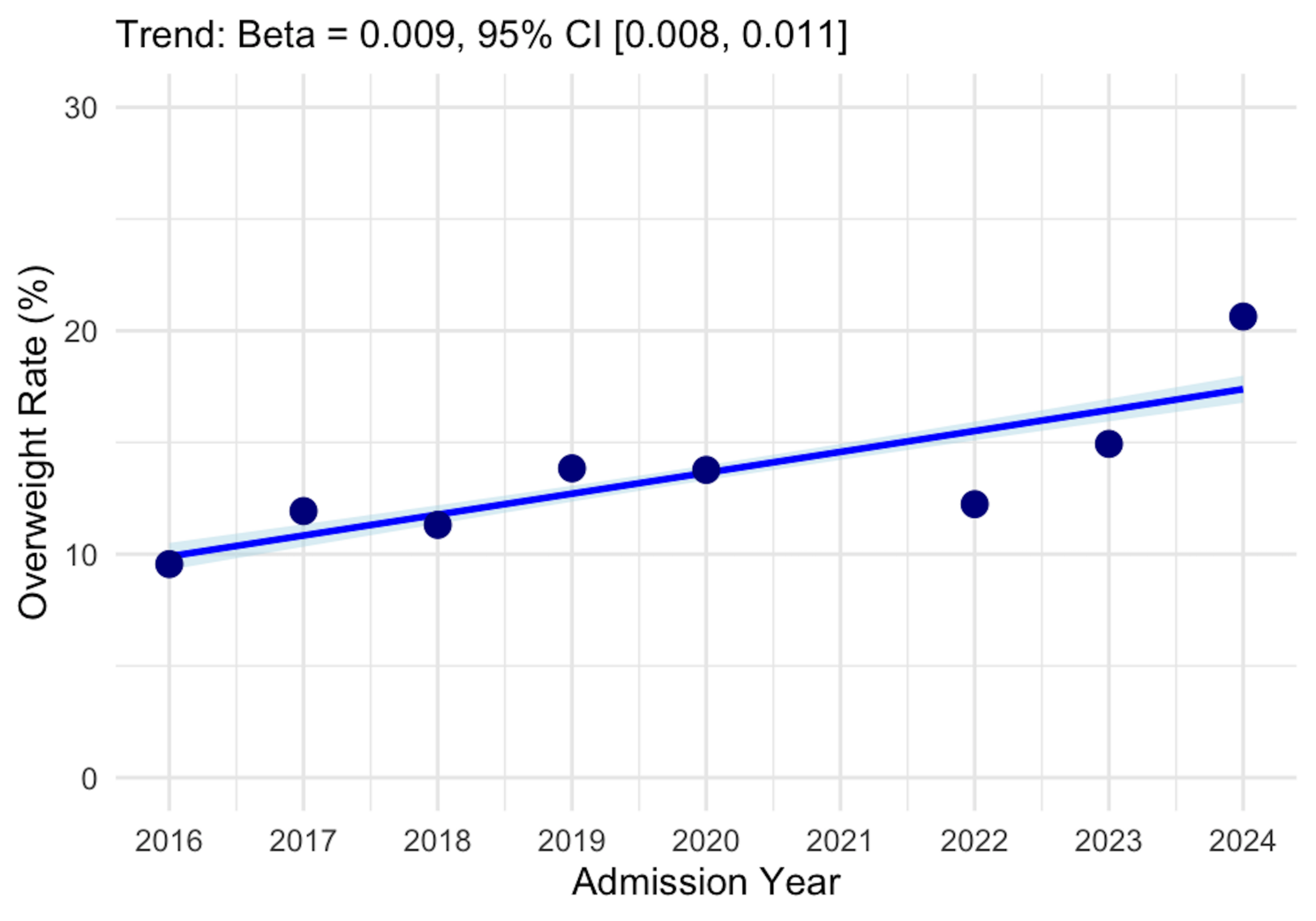
**

Abbreviations: CI, confidence interval.

(B) BMI ≥ 24 kg/m²


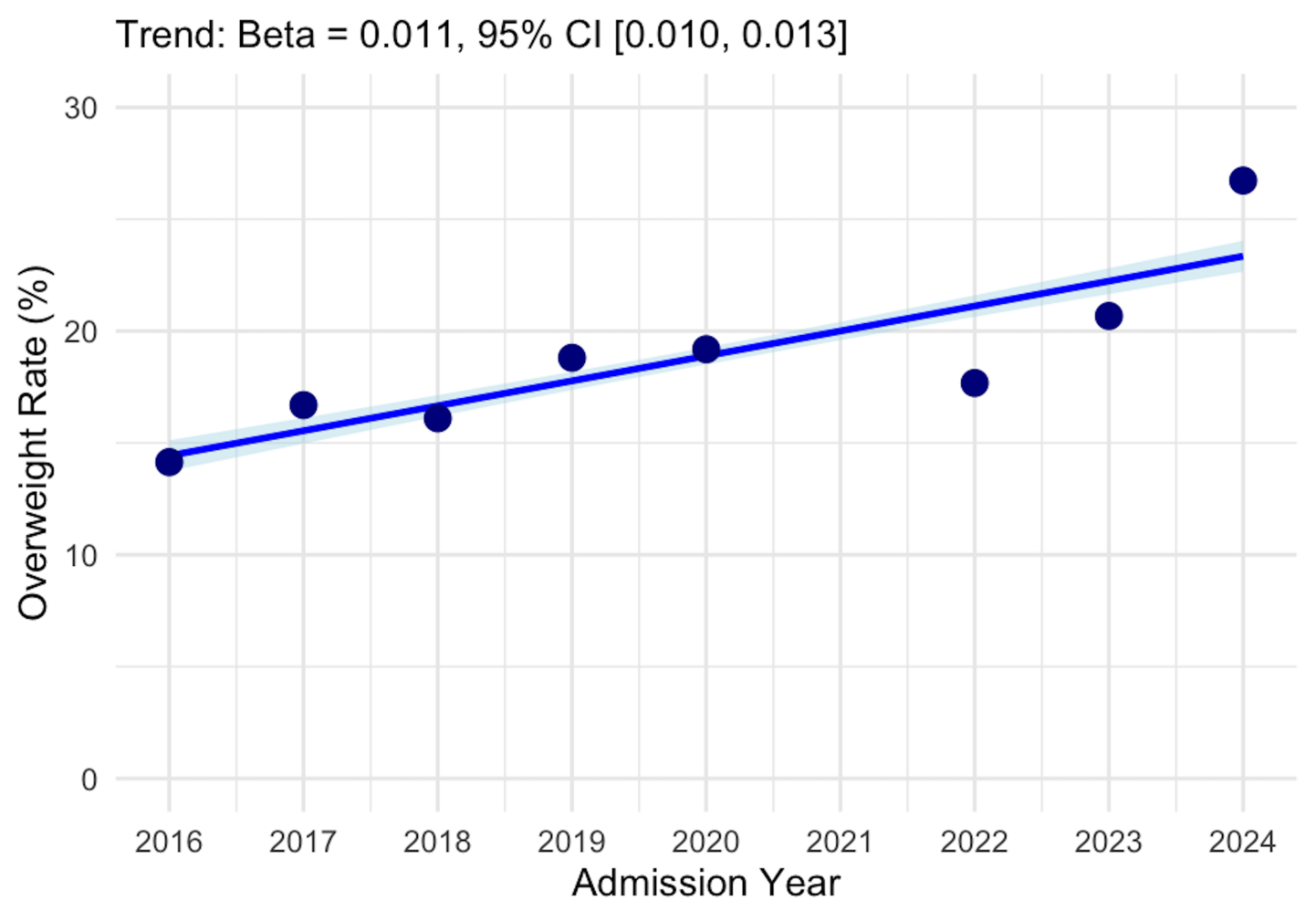


Abbreviations: CI, confidence interval.

**Supplementary Figure 3.** Nonlinear temporal trends in freshmen body mass index

(A) Result without imputation


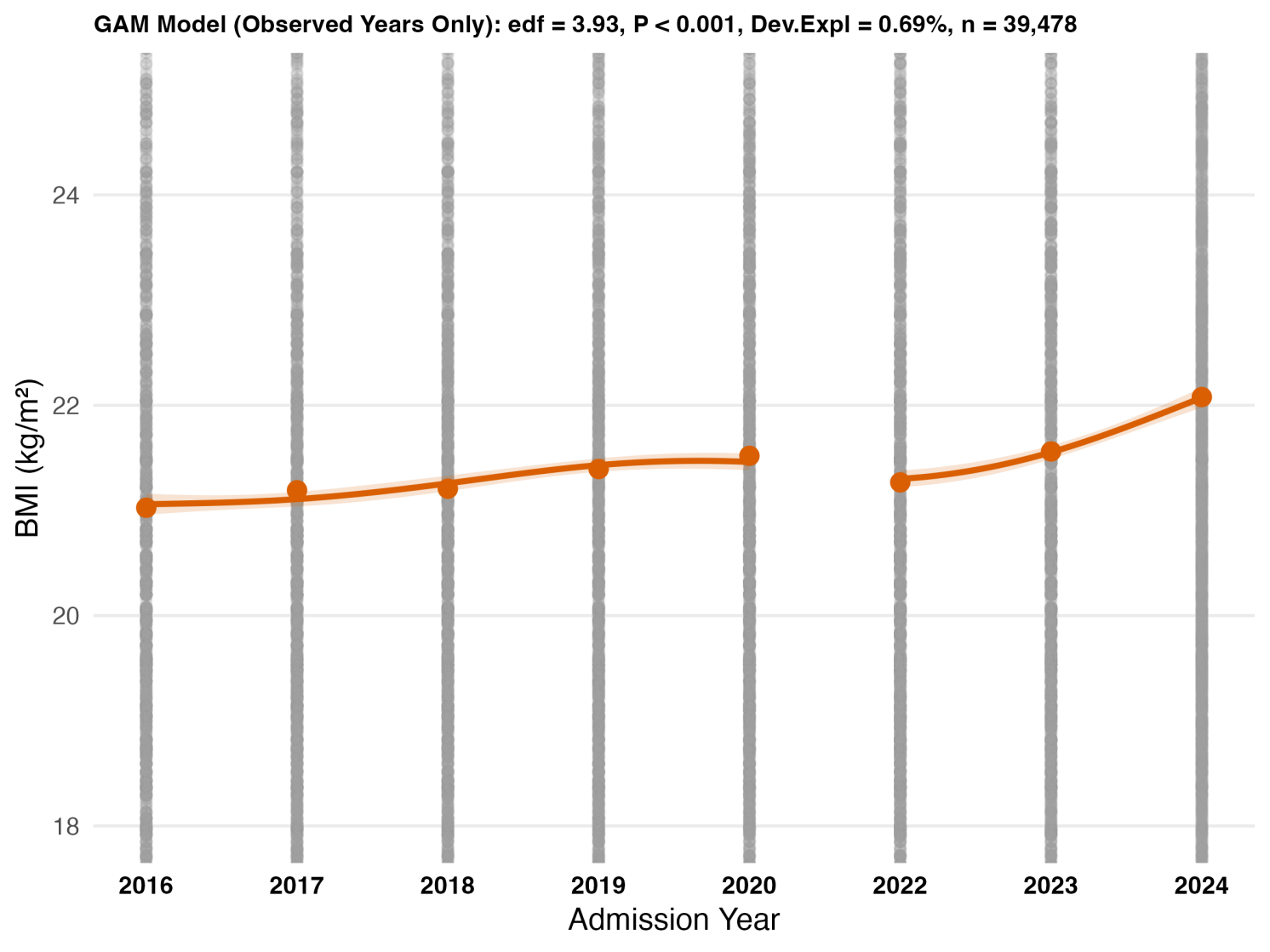


Abbreviations: BMI, body mass index; GAM, generalized additive model.

(B) Result with imputation


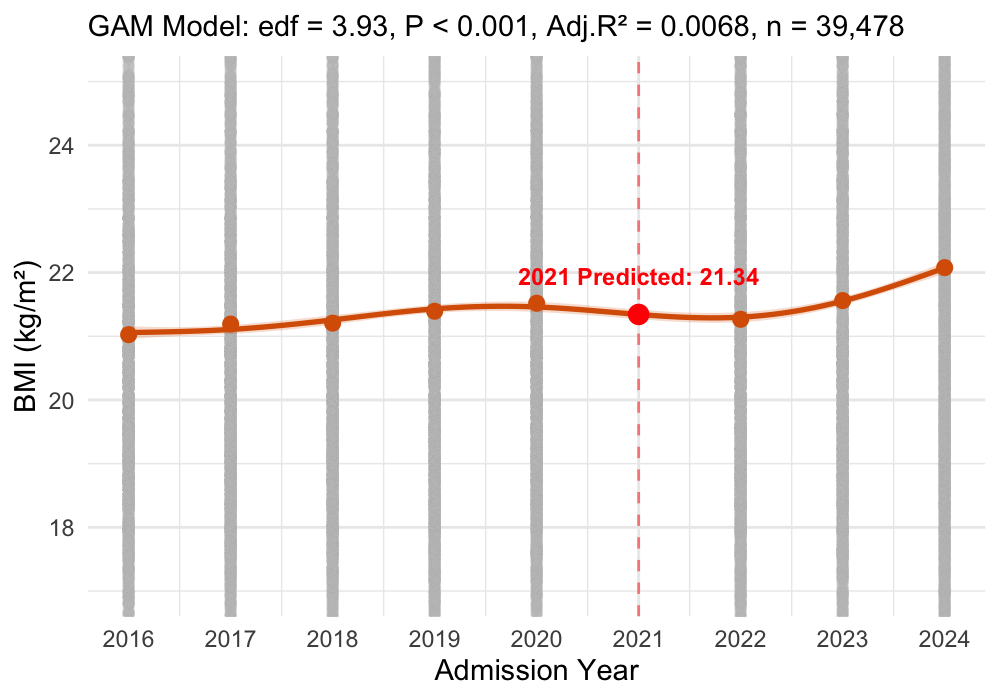


Abbreviations: BMI, body mass index; GAM, generalized additive model.

**Supplementary Figure 4.** Nonlinear temporal trends in overweight rate

(A) Result without imputation (BMI ≥ 25 kg/m²)


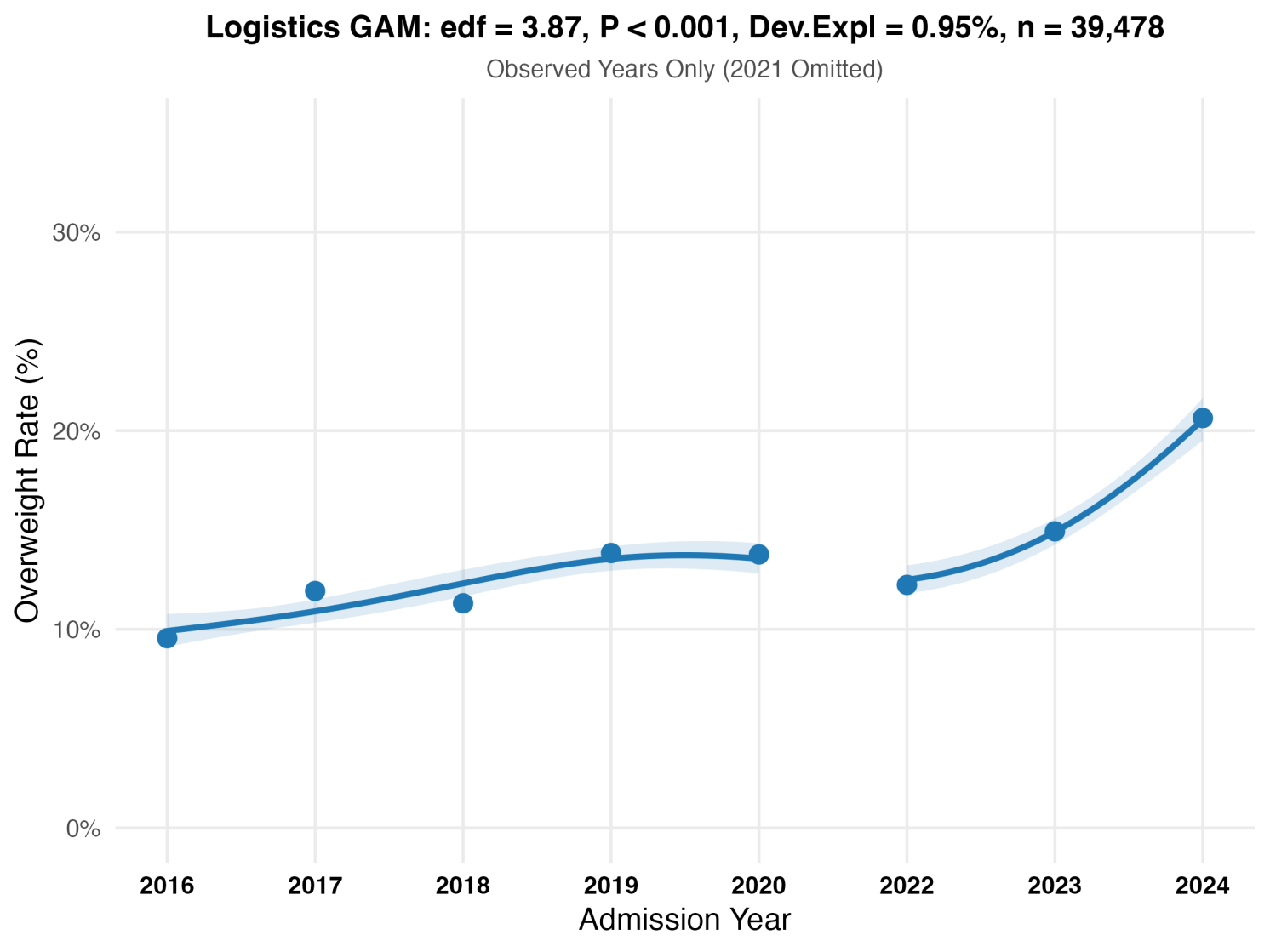


Abbreviations: GAM, generalized additive model.

(B) Result with imputation (BMI ≥ 25 kg/m²)


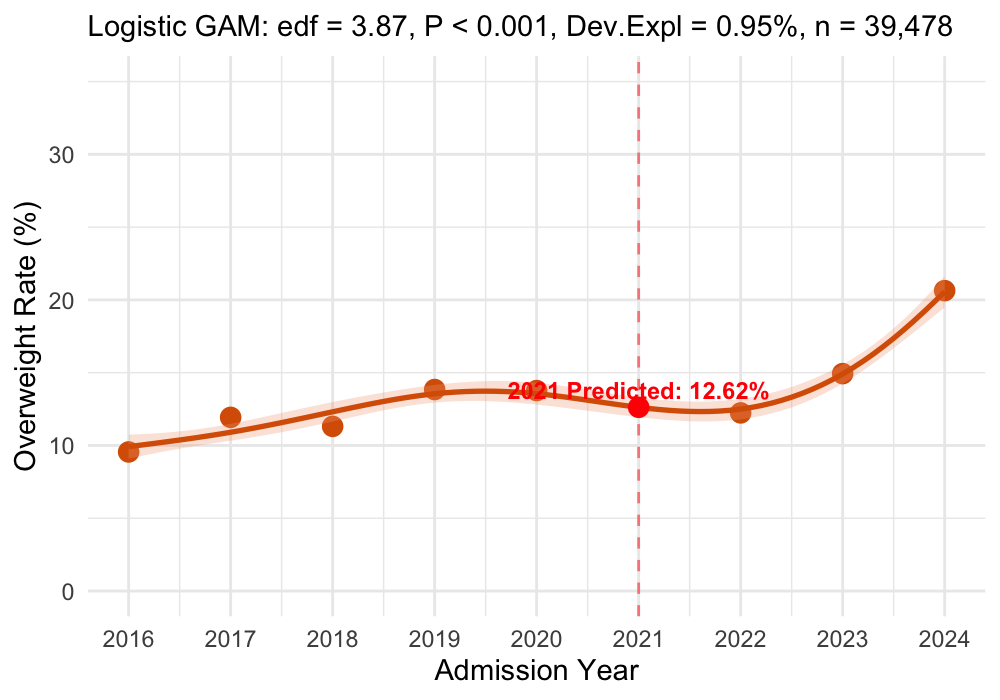


Abbreviations: GAM, generalized additive model.

(C) Result without imputation (BMI ≥ 24 kg/m²)


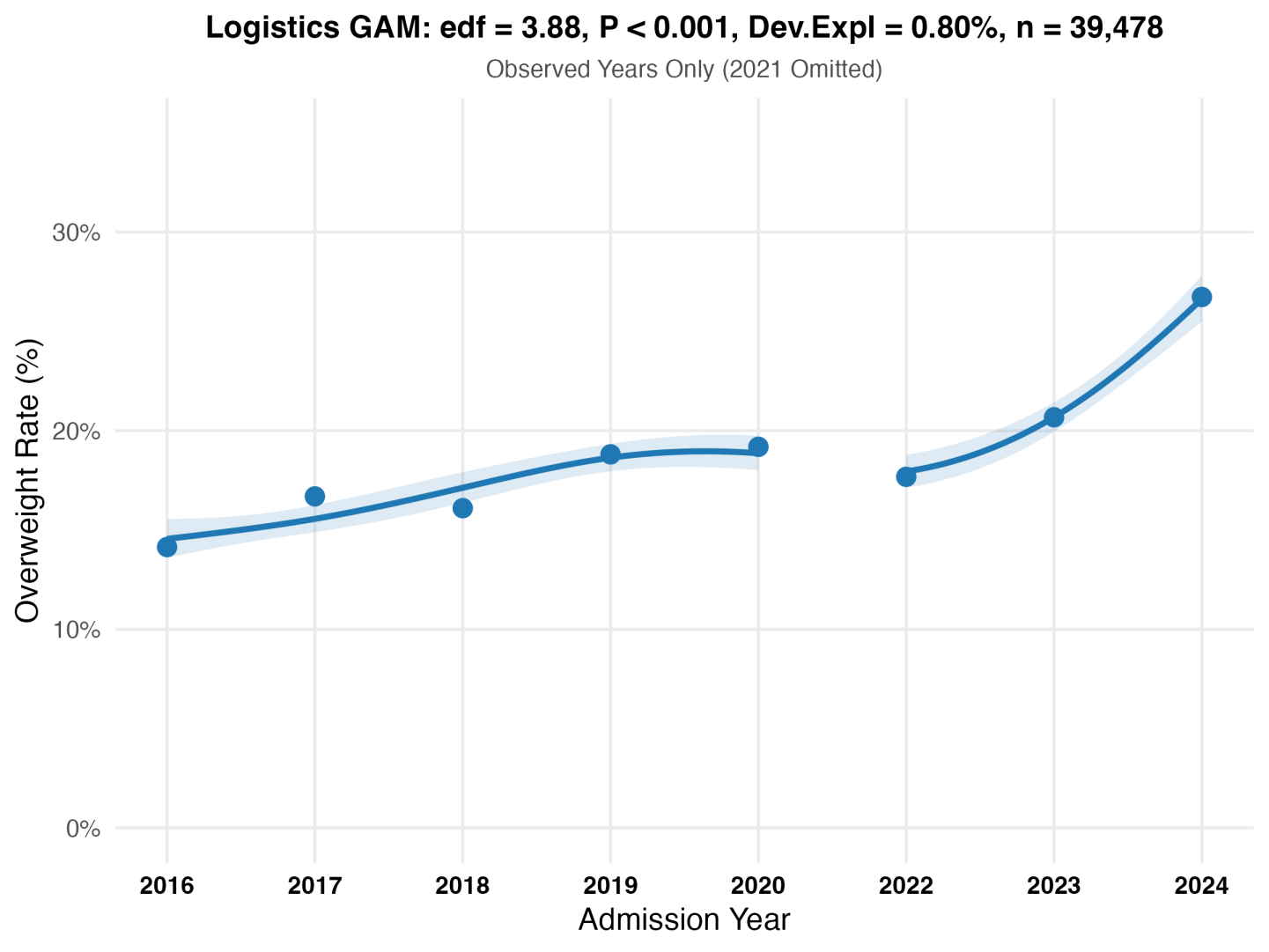


Abbreviations: GAM, generalized additive model.

(D) Result with imputation (BMI ≥ 24 kg/m²)


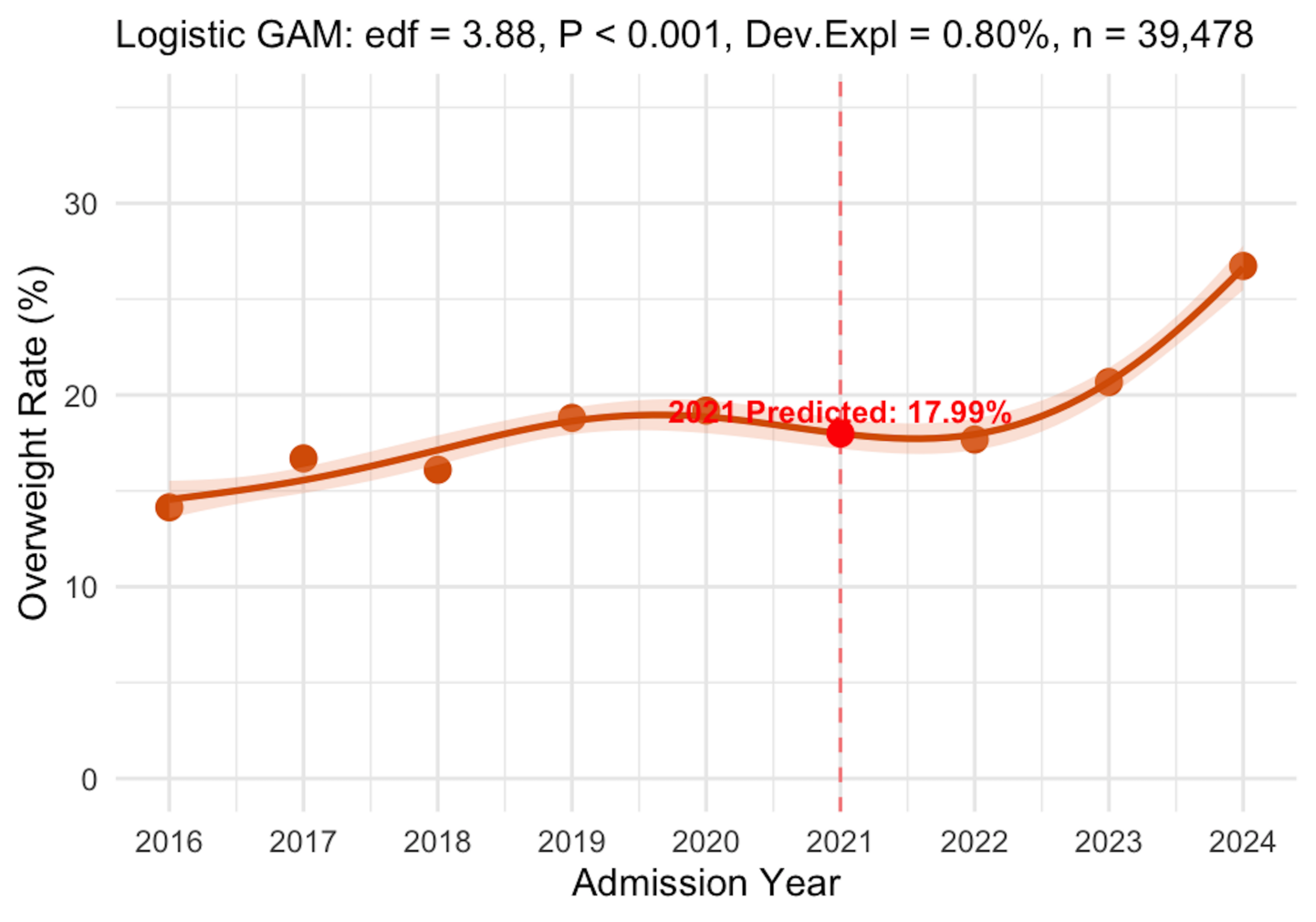


Abbreviations: GAM, generalized additive model.

**Supplementary Figure 5.** Interrupted time series analysis of mean BMI trends before and after COVID-19


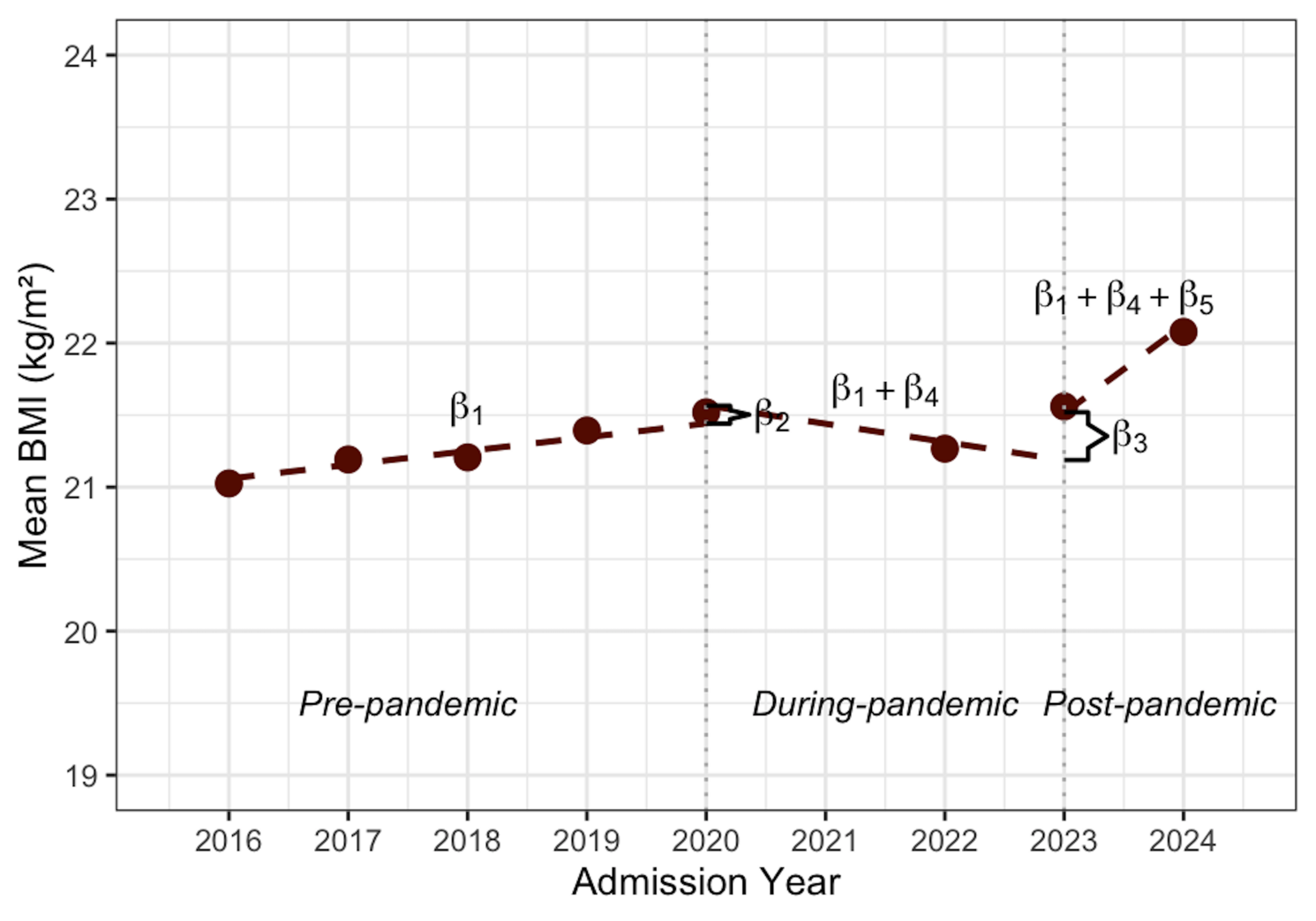


Abbreviations: BMI, body mass index.

Notes: Parameters β1 through β5 represent the estimated effects from the Interrupted Time Series (ITS) model:

·β1 (Baseline Trend): The annual change in BMI/Overweight rate during the pre-pandemic period (2016–2019).

·β2 (Step Change 2020): The immediate level shift in the outcome at the onset of the COVID-19 pandemic (2020).

·β3 (Step Change 2023): The immediate level shift in the outcome when the “Zero-COVID” policy ended (2023).

·β4 (Trend Change 1): The change in the annual slope during the pandemic period compared to the pre-pandemic trend.

·β5 (Trend Change 2): The change in the annual slope during the post-pandemic period compared to the during-pandemic trend.

**Supplementary Figure 6.** The temporal trends in overweight rate among the study participants using BMI ≥ 24 kg/m²

**
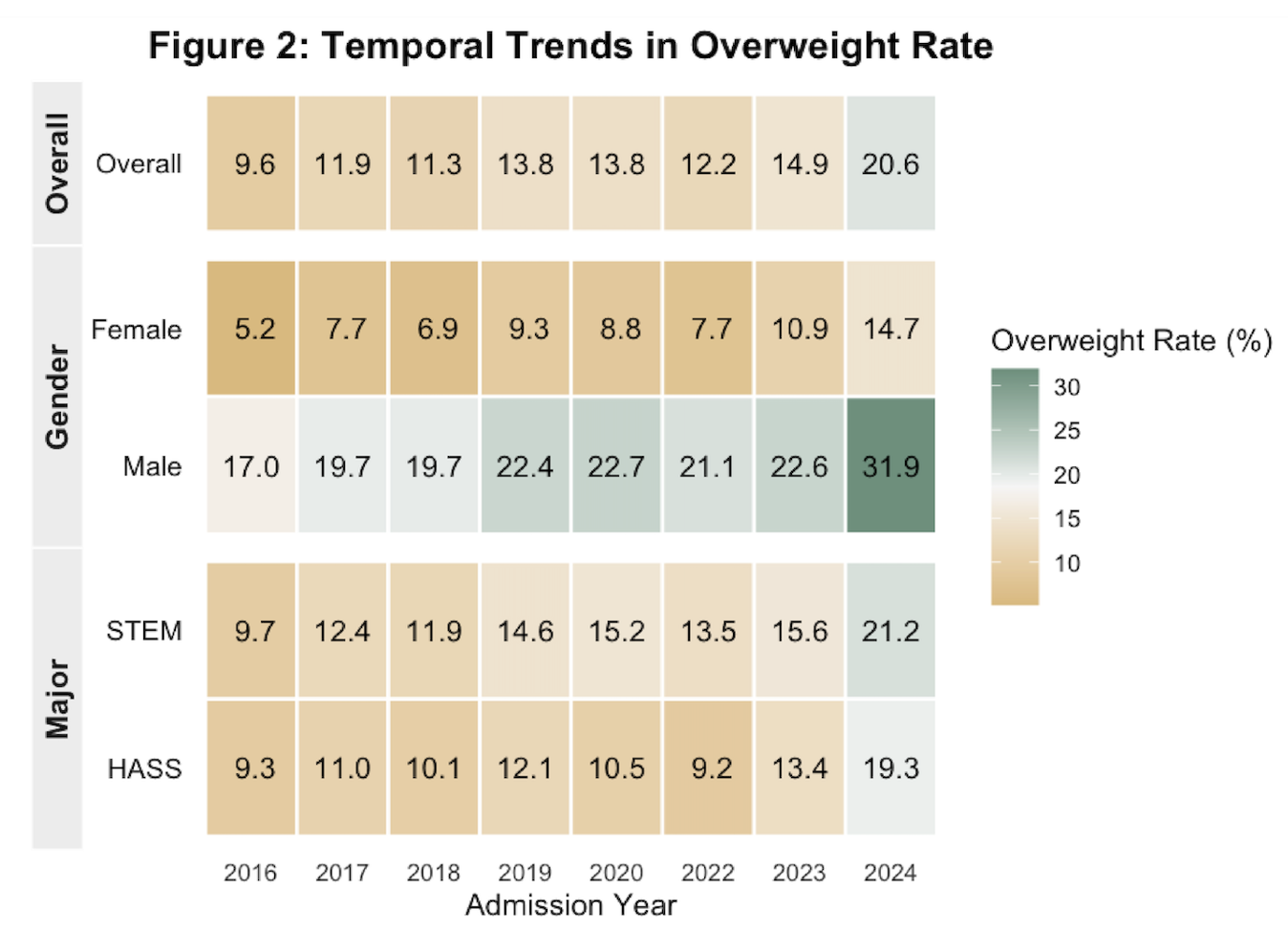
**

**Supplementary Figure 7.** Interrupted time series analysis of overweight rate trends before, during, and after COVID-19 using BMI ≥ 24 kg/m²

**
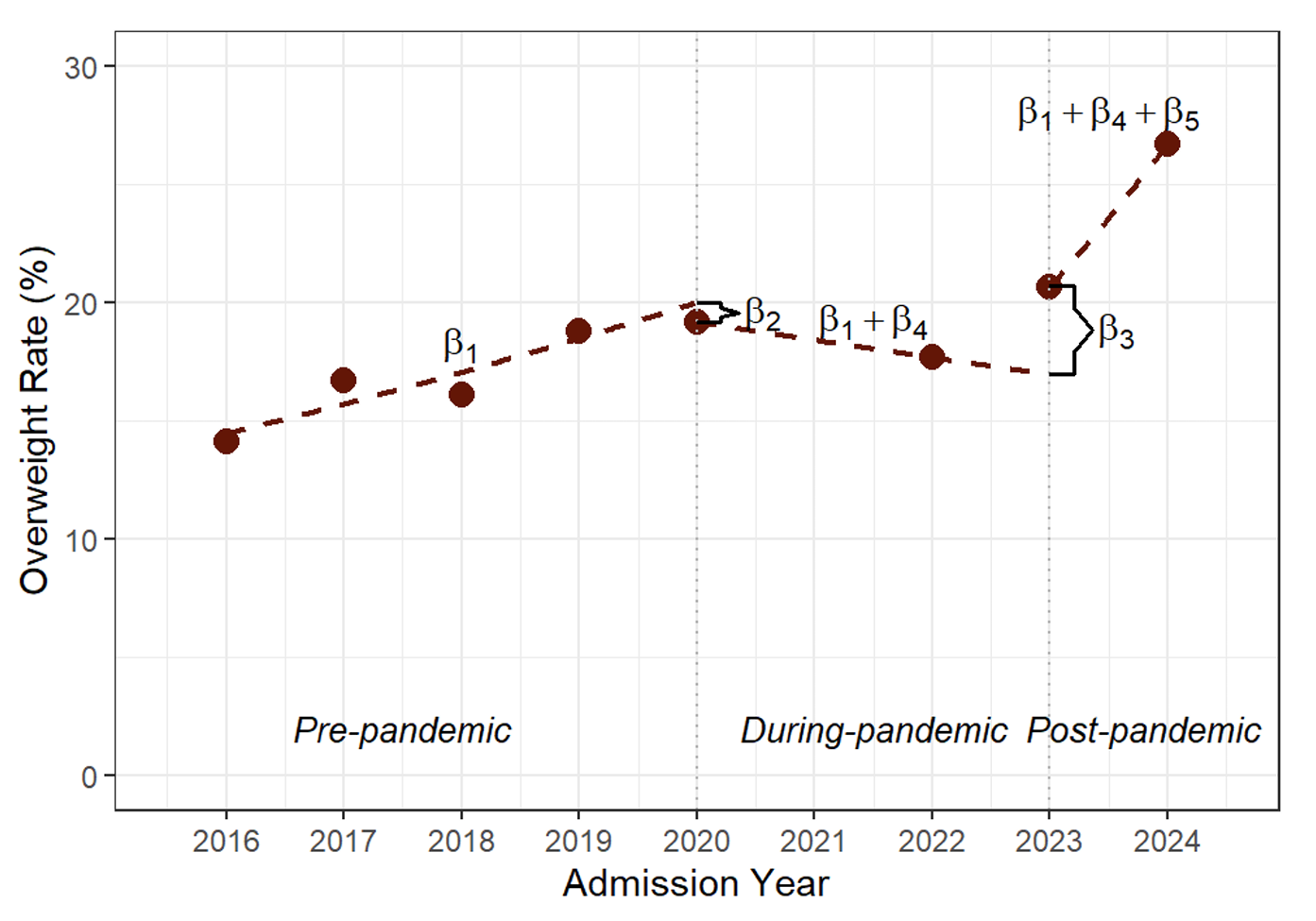
**

Abbreviations: BMI, body mass index.

Notes: Parameters β1 through β5 represent the estimated effects from the Interrupted Time Series (ITS) model:

·β1 (Baseline Trend): The annual change in BMI/Overweight rate during the pre-pandemic period (2016–2019).

·β2 (Step Change 2020): The immediate level shift in the outcome at the onset of the COVID-19 pandemic (2020).

·β3 (Step Change 2023): The immediate level shift in the outcome when the “Zero-COVID” policy ended (2023).

·β4 (Trend Change 1): The change in the annual slope during the pandemic period compared to the pre-pandemic trend.

·β5 (Trend Change 2): The change in the annual slope during the post-pandemic period compared to the during-pandemic trend.

**Supplementary Figure 8.** Interrupted time series analysis of mean BMI trends before, during, and after COVID-19 by sex

**
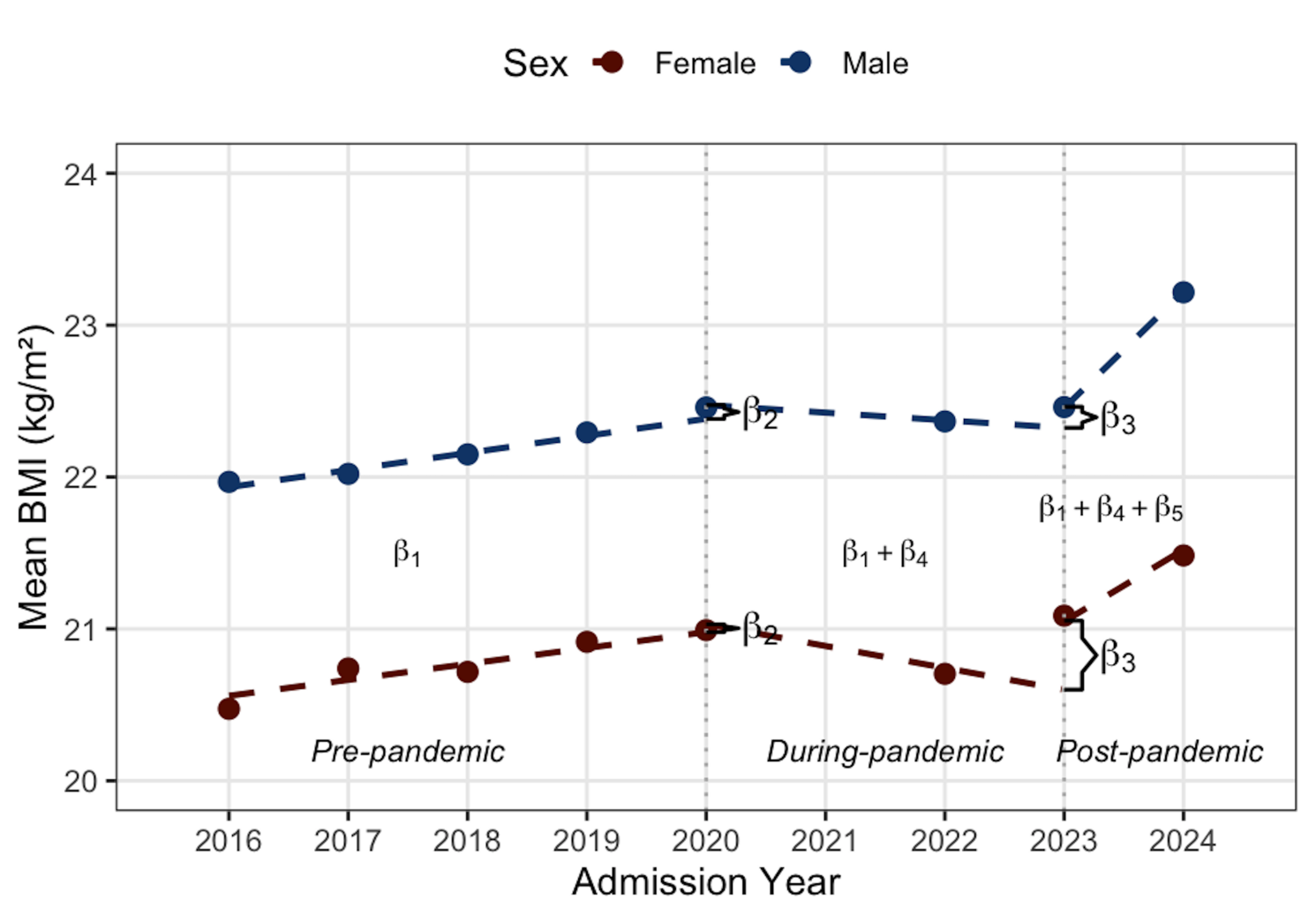
**

Abbreviations: BMI, body mass index.

Notes: Parameters β1 through β5 represent the estimated effects from the Interrupted Time Series (ITS) model:

·β1 (Baseline Trend): The annual change in BMI/Overweight rate during the pre-pandemic period (2016–2019).

·β2 (Step Change 2020): The immediate level shift in the outcome at the onset of the COVID-19 pandemic (2020).

·β3 (Step Change 2023): The immediate level shift in the outcome when the “Zero-COVID” policy ended (2023).

·β4 (Trend Change 1): The change in the annual slope during the pandemic period compared to the pre-pandemic trend.

·β5 (Trend Change 2): The change in the annual slope during the post-pandemic period compared to the during-pandemic trend.

**Supplementary Figure 9.** Interrupted time series analysis of overweight rate trends before, during, and after COVID-19 by sex

(A) BMI ≥ 25 kg/m²

**
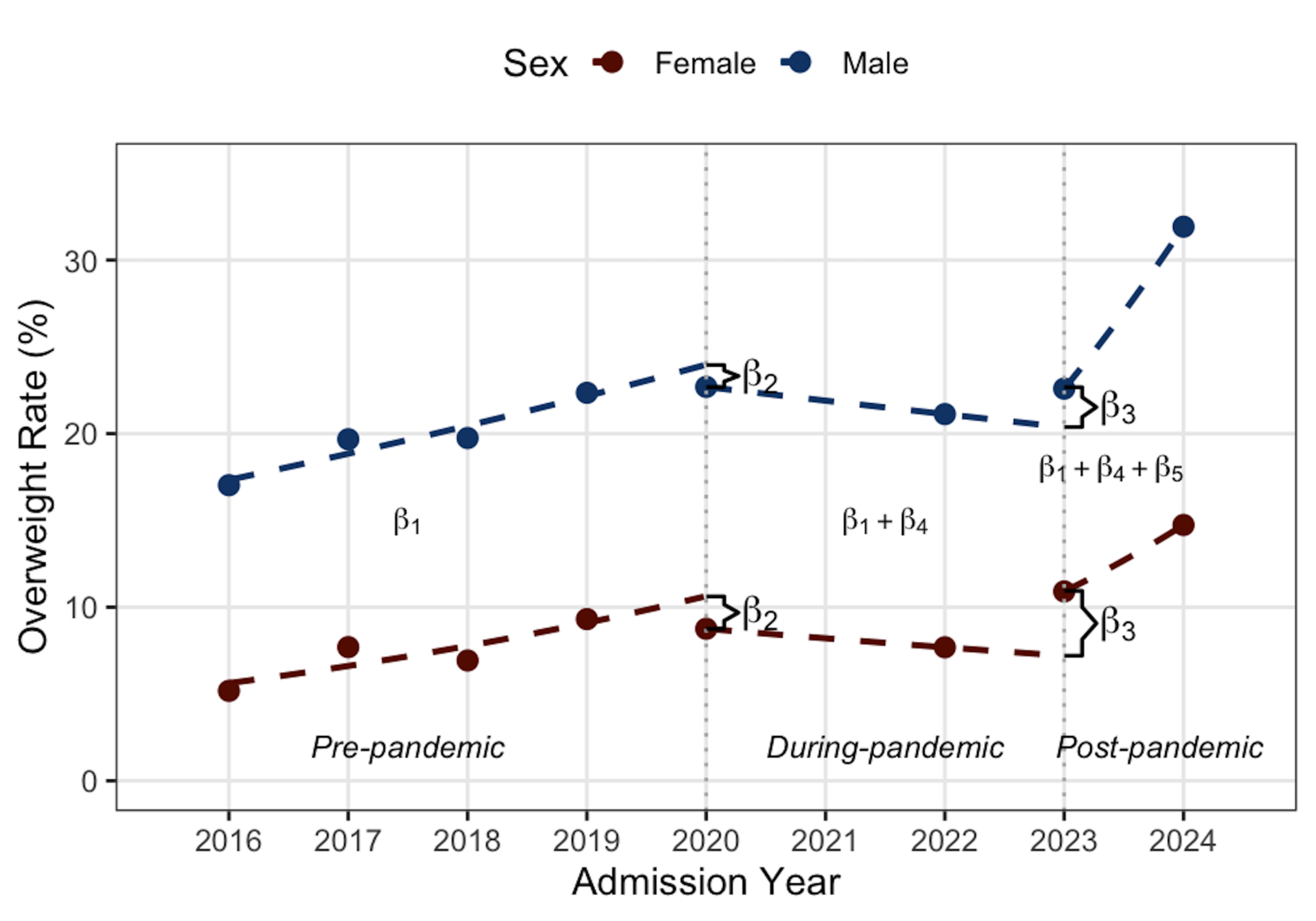
**

(B) BMI ≥ 24 kg/m²


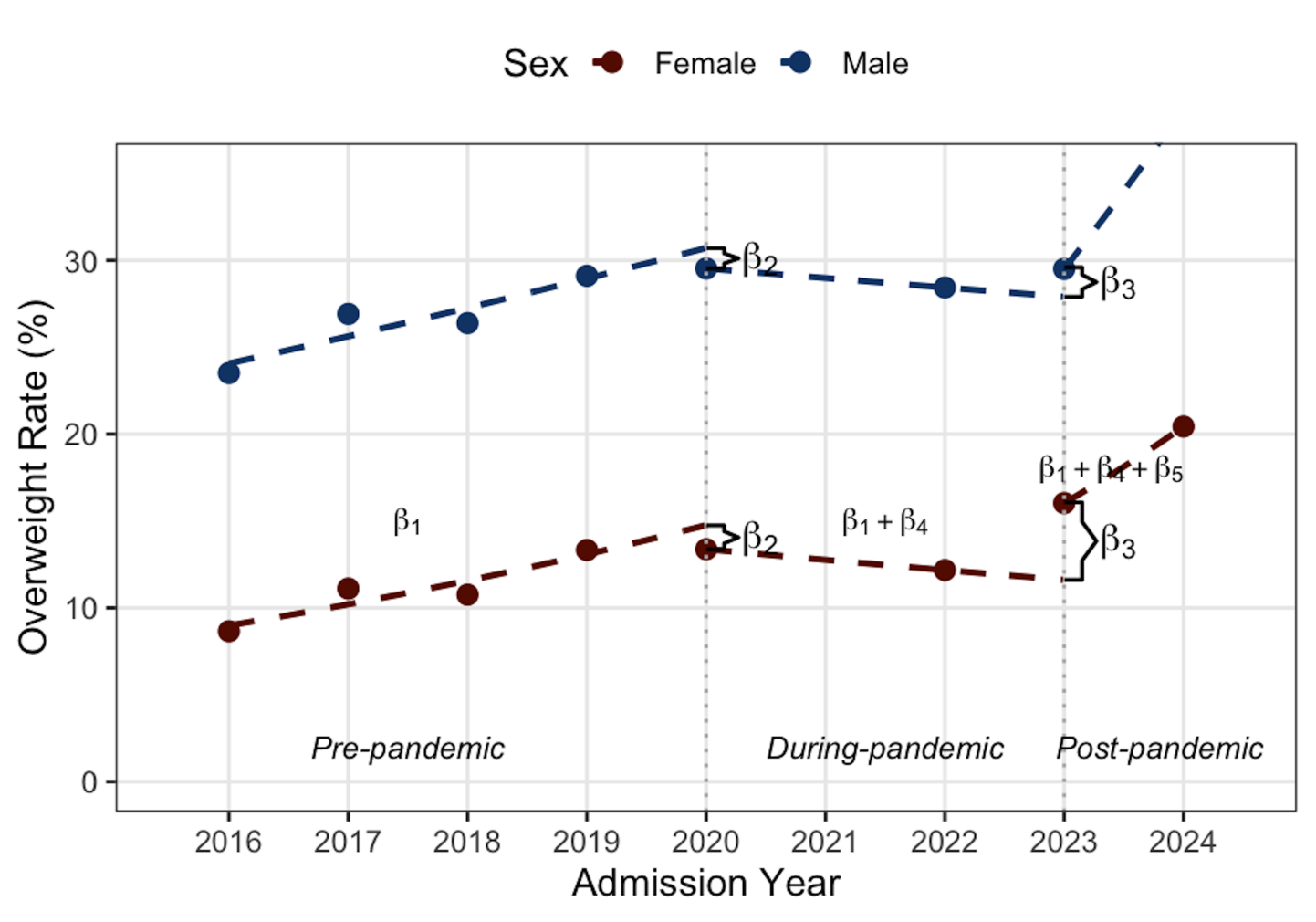


Notes: Parameters β1 through β5 represent the estimated effects from the Interrupted Time Series (ITS) model:

·β1 (Baseline Trend): The annual change in BMI/Overweight rate during the pre-pandemic period (2016–2019).

·β2 (Step Change 2020): The immediate level shift in the outcome at the onset of the COVID-19 pandemic (2020).

·β3 (Step Change 2023): The immediate level shift in the outcome when the “Zero-COVID” policy ended (2023).

·β4 (Trend Change 1): The change in the annual slope during the pandemic period compared to the pre-pandemic trend.

·β5 (Trend Change 2): The change in the annual slope during the post-pandemic period compared to the during-pandemic trend.

**Supplementary Figure 10.** Interrupted time series analysis of mean BMI trends before, during, and after COVID-19 by discipline


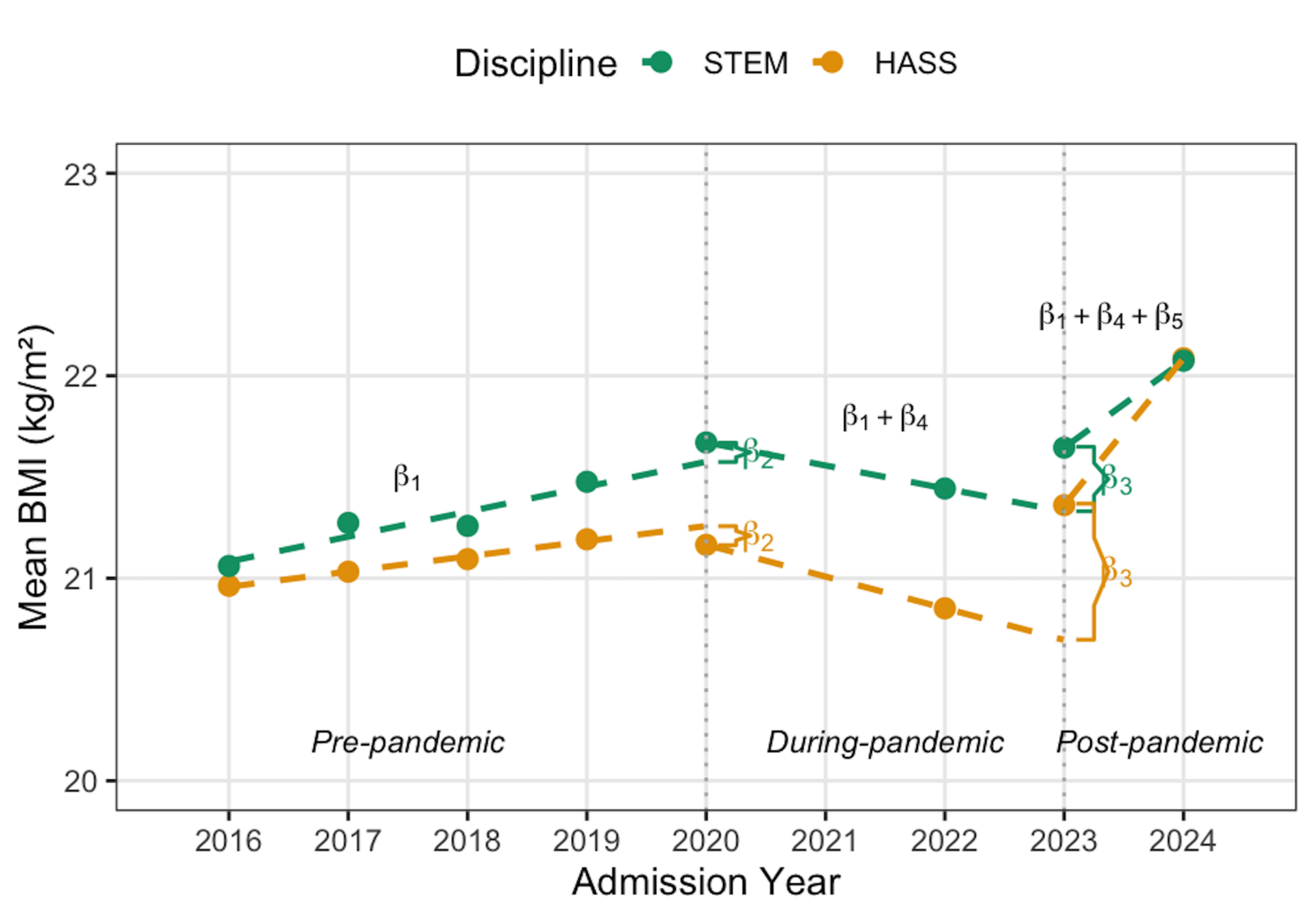


Abbreviations: BMI, body mass index.; STEM, science, technology, engineering, and mathematics; HASS, humanities, arts and social sciences.

Notes: Parameters β1 through β5 represent the estimated effects from the Interrupted Time Series (ITS) model:

·β1 (Baseline Trend): The annual change in BMI/Overweight rate during the pre-pandemic period (2016–2019).

·β2 (Step Change 2020): The immediate level shift in the outcome at the onset of the COVID-19 pandemic (2020).

·β3 (Step Change 2023): The immediate level shift in the outcome when the “Zero-COVID” policy ended (2023).

·β4 (Trend Change 1): The change in the annual slope during the pandemic period compared to the pre-pandemic trend.

·β5 (Trend Change 2): The change in the annual slope during the post-pandemic period compared to the during-pandemic trend.

**Supplementary Figure 11.** Interrupted time series analysis of overweight rate trends before, during, and after COVID-19 by discipline

(A) BMI ≥ 25 kg/m²


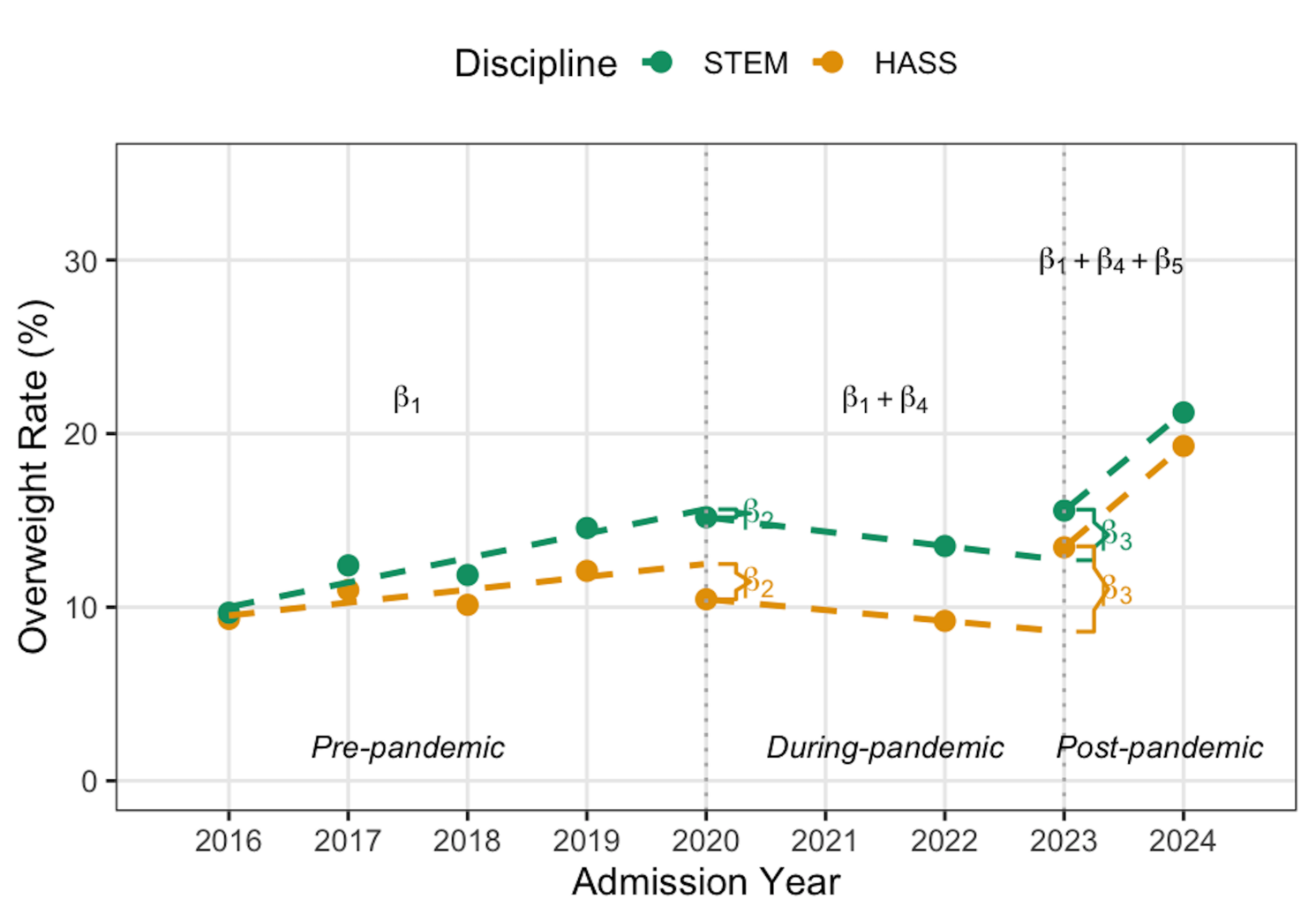


Abbreviations: STEM, science, technology, engineering, and mathematics; HASS, humanities, arts and social sciences.

(B) BMI ≥ 24 kg/m²


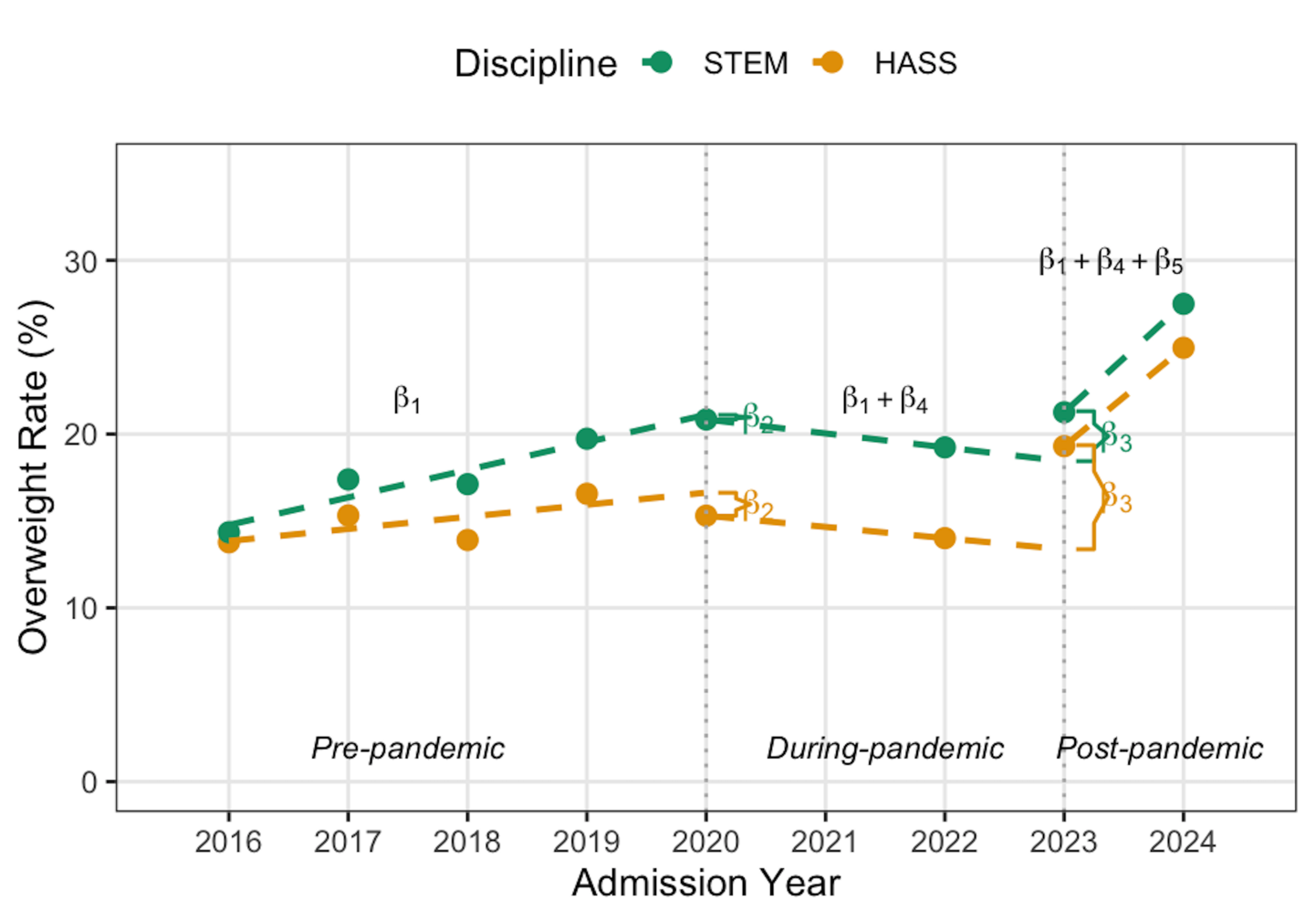


Abbreviations: STEM, science, technology, engineering, and mathematics; HASS, humanities, arts and social sciences.

Notes: Parameters β1 through β5 represent the estimated effects from the Interrupted Time Series (ITS) model:

·β1 (Baseline Trend): The annual change in BMI/Overweight rate during the pre-pandemic period (2016–2019).

·β2 (Step Change 2020): The immediate level shift in the outcome at the onset of the COVID-19 pandemic (2020).

·β3 (Step Change 2023): The immediate level shift in the outcome when the “Zero-COVID” policy ended (2023).

·β4 (Trend Change 1): The change in the annual slope during the pandemic period compared to the pre-pandemic trend.

·β5 (Trend Change 2): The change in the annual slope during the post-pandemic period compared to the during-pandemic trend.
